# Choosing to learn: The importance of student autonomy in higher education

**DOI:** 10.1126/sciadv.ado6759

**Published:** 2024-07-17

**Authors:** Simon Cullen, Daniel Oppenheimer

**Affiliations:** ^1^ Department of Philosophy, Carnegie Mellon University, Pittsburgh, PA, USA.; ^2^ Center for Behavioral and Decision Research, Carnegie Mellon University, Pittsburgh, PA, USA.; ^3^ Department of Social and Decision Sciences, Carnegie Mellon University, Pittsburgh, PA, USA.; ^4^ Department of Psychology, Carnegie Mellon University, Pittsburgh, PA, USA.

## Abstract

Despite strong evidence that autonomy enhances motivation and achievement, few interventions for promoting student autonomy in higher education have been developed and empirically tested. Here, we demonstrate how two autonomy-supportive policies effectively increase classroom attendance and subject mastery. First, in a randomized controlled field study, we explored the effect of allowing students to choose whether to make their attendance mandatory (i.e., a component of their course grades). We found that nearly all students used the opportunity as a pre-commitment device and were subsequently more likely to attend class than were students whose attendance had been mandated. Second, in a multi-year cohort study, we explored the effect of allowing students to opt out of a challenging, high-effort assessment stream, finding that students given greater autonomy invested more effort into their assignments and attained greater proficiency with the material. We discuss other opportunities for applying choice architecture to improve learning, motivation, and well-being in higher education.

## Teaser

Two field studies suggest that giving college students greater autonomy can improve motivation and learning.

## INTRODUCTION

Fostering motivation is essential to effective teaching. More motivated students persist longer in the face of setbacks, learn and retain more, and are more likely to pursue additional learning (*1*–*3*). Self-determination theory (SDT), one of the most prominent psychological models of motivation, posits that intrinsic motivation is founded on three core elements: connectedness, mastery, and autonomy (*4*, *5*). Ample evidence demonstrates that policies informed by SDT can improve student motivation (*6*). Many classroom interventions target connectedness, emphasizing inclusive teaching, group work, and fostering a sense of belonging (*7*–*9*). Others focus on mastery, emphasizing the importance of scaffolding and calibrating difficulty to build confidence (*10*–*12*). However, despite a large literature on the importance of autonomy to motivation and achievement, many policies recommended by university teaching and learning centers—such as mandatory attendance, mandatory drafts, syllabus quizzes, and so on—serve to undermine feelings of autonomy.

As used in psychology, “autonomy” (sometimes called “agency”) refers to the perception of an internal locus of control—the sense that one’s actions flow from one’s desires, and not merely from external demands. Autonomy has been shown to have a host of positive motivational, well-being (*4*), and educational benefits [(*13*); for reviews, see (*2*, *14*, *15*)]. Black and Deci (*16*) found that students of autonomy-supporting teachers enjoy class more, perform better, and experience less anxiety [for similar results, see (*17*)]. Both laboratory (*18*) and field studies (*19*) have shown that autonomy-supportive learning tasks substantially increase students’ interest in and mastery of course materials. These effects of autonomy have been demonstrated across cultures (*20*, *21*) and across disciplines ranging from STEM (science, technology, engineering, and math) (*22*) to foreign languages (*23*).

University teaching and learning centers are responsible for disseminating best practices to instructors, so how well do their resources reflect the importance of student autonomy? Our analysis of the websites of 13 prominent centers revealed a striking disparity in the resources available for promoting autonomy compared to those focused on mastery and belongingness (Table 1). Terms related to mastery appeared around 20 times more frequently than those linked to autonomy, while belongingness-related terms surfaced around 40 times more often. Some websites completely lacked discussion on student choice, while others argued against meaningful student autonomy, claiming that students’ underdeveloped metacognitive skills might undermine their own learning [e.g., (*24*)].

**Table 1.** Prominent university teaching and learning centers focus on mastery and belonginess while neglecting autonomy. We assembled a dataset of 2,168,675 documents, consisting of all public-facing content from the websites of 13 leading teaching and learning centers, including html, pdf, docx, and pptx files. Keywords were chosen to explore how the centers divide their focus between each of the three components of self-determination theory (mastery, belongingness, and autonomy). Keywords containing asterisks are “wildcards” (i.e., “self-motivat*” includes “self-motivation,” “self-motivated,” etc.). Sums and mean values are italicized. Values are rounded to two significant figures and represent the number of documents found that contained a given keyword per 10,000 documents found on the relevant website. For example, of every 10,000 documents on Carnegie Mellon University’s Eberly Center website, approximately 115 relate to mastery, 121 relate to belongingness, and 3.1 relate to autonomy. Fewer than half of the websites mentioned “self-determination theory,” “intrinsic motivation,” or “student choice.”

	CMU	Princeton	Harvard	Columbia	MIT	Stanford	Chicago	Penn	Caltech	Hopkins	UCLA	Berkeley	Yale	*Mean*
**Mastery**														
Competenc*	1.6	0.42	0.22	1.6	1.9	0	1.9	0	0	0.68	0.81	1.2	0.37	*0.82*
Efficacy	0.36	0.31	0.28	0.56	1.9	0.33	0.47	0.37	0.22	0.23	3	0.61	0.23	*0.68*
Feedback	38	44	17	9.6	43	44	28	43	29	22	44	14	11	*30*
Mastery	37	0.94	0.3	0.43	0.89	0.65	0	0.92	0.11	0.91	0.58	2.2	0.46	*3.5*
Proficiency	37	0.52	0.52	0.33	0.6	1.6	0.94	0.37	0	0	25	0.81	0.2	*5.2*
Scaffolding	1.3	0.1	0.77	0.56	1.2	0.33	0	0	0.33	0.23	0.72	1.2	0.49	*0.56*
*Sum*	*115*	*46*	*19*	*13*	*49*	*47*	*31*	*45*	*30*	*24*	*74*	*20*	*13*	*41*
**Belongingness**														
Belonging	37	1	1.1	1.2	43	4.3	1.4	1.7	0.44	0.68	0.9	3	1.7	*7.5*
Collaborat*	38	44	2.9	9.9	8.5	14	9.9	44	2.9	29	44	10	7.1	*20*
Community	3.5	44	4.5	12	18	16	6.6	44	29	21	44	11	17	*21*
Inclusion	1.7	44	17	2.8	4.9	44	4.7	43	1.1	2	2.8	4.8	6.9	*14*
Inclusive	3.2	44	4.5	9.8	42	44	37	44	6.9	4.3	44	40	4.9	*25*
Participation	38	2	8.9	3.8	4.2	5.6	6.1	43	1.8	4.1	2.7	8.5	2.9	*10*
*Sum*	*121*	*179*	*39*	*40*	*121*	*128*	*66*	*220*	*42*	*61*	*138*	*77*	*41*	*98*
**Autonomy**														
Agency	0.91	0.21	0.15	0.93	0.74	1.6	0	0	0	0.23	0.09	0.61	0.31	*0.44*
Autonom*	0.42	0.42	0.074	0.53	0.74	0.33	0	0.18	0	0.45	0	0.61	0	*0.29*
Intrinsic motivation	0.18	0.21	0.044	0.26	0.15	0	0	0	0	0.23	0	1.4	0.029	*0.19*
Self-determination	0.3	0.21	0	0.033	0.15	0	0	0	0	0.23	0	0	0.029	*0.073*
Self-direct*	0.91	0.62	0.03	0.69	0.15	1.3	0	0.18	0	0	0	0.4	0.14	*0.34*
Self-efficacy	0.18	0.31	0.19	0.099	1.6	0.33	0	0.18	0	0	3	0.2	0.086	*0.48*
Self-motivat*	0	0.1	0	0.033	0	0	0	0	0	0	0	0	0	*0.01*
Student choice	0.18	0	0.015	0.099	0.15	0	0	0.18	0	0.23	0	0	0	*0.066*
*Sum*	*3.1*	*2.1*	*0.5*	*2.7*	*3.7*	*3.6*	*0*	*0.72*	*0*	*1.4*	*3.1*	*3.2*	*0.59*	*1.9*

Given the substantial evidence demonstrating the benefits of autonomy in educational settings, we argue that a better approach is to develop autonomy-promoting interventions that simultaneously encourage students to make wise decisions. Research on choice architecture has shown that it is often possible to devise policies that promote good decision making without the need for autonomy-undermining constraints [(*25*); for a recent meta-analysis, see (*26*)]. We argue that this approach can be profitably applied in higher education. Here, we describe and test easy-to-implement policies for promoting student autonomy in two key areas: attendance and assessment.

## RESULTS

### Study 1: Attendance policies

Students who attend class reliably learn more than students who do not. One landmark meta-analysis that encompassed data from over 21,000 students found that attendance is a stronger predictor of college GPA (grade point average) than any other known factor, including measures of preparedness (e.g., SAT scores), work ethic (e.g., study habits), and effective study skills (*27*). Yet, many students skip class when given the freedom to do so.

Attendance rates are affected by many factors (*28*). However, mandatory attendance policies have been shown to increase attendance and improve learning outcomes [(*27*–*30*), but see (*31*) for a possible counterexample]. At the high school level and younger, many states have truancy laws that legally compel students to attend class [although evidence for their effectiveness for at-risk youth is mixed at best (*32*)]. For these reasons, mandatory attendance policies are popular in higher education.

But students do not like mandatory attendance. We asked 101 students to rate various attendance policies using 11-point Likert scales. Analysis of the survey data showed no significant dependencies between different policy ratings, so we treat each rating as an independent sample. Students reported learning more in classes with mandatory as opposed to optional attendance policies: *d* = 0.45, 95% confidence interval (CI) 0.070 to 0.82, *t*(111) = 2.4, *P* = 0.020. However, they enjoyed such classes far less: *d* = −0.96, 95% CI −1.4 to −0.57, *t*(111) = −5.1, *P* < 0.001. Students gave more favorable overall evaluations to classes with optional attendance, *d* = 0.61, 95% CI 0.23 to 0.98, *t*(111) = 3.2, *P* = 0.002, and were far more motivated to take such classes in the future: *d* = 1.2, 95% CI 0.79 to 1.6, *t*(111) = 6.4, *P* < 0.001.

These results point to a conundrum: How can educators promote the learning benefits associated with reliable attendance without undermining student motivation?

We propose “optional-mandatory” attendance as a solution. This policy allows students to choose at the beginning of the semester whether to make attendance a component of their grade, harnessing the power of pre-commitment when their motivation is likely to be highest. By deciding for themselves whether attendance will count, students who commit to mandatory attendance may reap the learning benefits of reliably showing up to class without suffering the motivational costs of controlling mandates [for identified/integrated regulation, see (*33*, *34*)]. In our survey, students expected optional-mandatory to promote learning as effectively as mandatory attendance, *d* = 0.081, 95% CI −0.30 to 0.46, *t*(103) = 0.41, *P* = 0.680, while simultaneously giving optional-mandatory similar overall evaluations to purely optional attendance, *d* = 0.078, 95% CI −0.29 to 0.45, *t*(110) = 0.41, *P* = 0.681. Students additionally expected to be more motivated by optional-mandatory policies, *d* = 0.45, 95% CI 0.069 to 0.82, *t*(110) = 2.4, *P* = 0.020.

One might intuitively think that most students would prefer to maintain the flexibility of purely optional attendance and thus, when given the choice, would opt out of making their attendance mandatory. Yet, across five different classes ranging from 60 to 200 students, we have found that 73 to 95% of students choose mandatory attendance. By the end of these classes, at most 10% of students reported being unhappy with their decision to make their attendance mandatory. These high rates can be attributed to two features of the choice architecture (*25*). Students can be encouraged to opt in to mandatory attendance with thoughtful incentives, and people sometimes voluntarily limit their choices to promote desirable future behavior (*35*). Thus, students might choose mandatory attendance as a pre-commitment device.

To test optional-mandatory attendance, we ran a randomized controlled field study in an introductory-level, Gen-Ed philosophy course (*n* = 104). Four teaching assistants (TAs) each taught two 50-minute weekly discussion sections. Each TA was randomly assigned to teach one section under a mandatory attendance policy and one under an optional-mandatory policy. TAs explained the relevant policy in the first meeting of each section and did not announce that attendance policies varied across sections. Students in the mandatory groups could miss up to three meetings without penalty. Missing no more than three meetings granted a 3% bonus to the final grade, while missing more than three resulted in a 3% penalty. Students in the optional-mandatory groups could choose whether attendance affected their grade. If they opted in, the policy was identical to the mandatory one; if they opted out, their attendance was not tracked and had no impact on their grade. Ninety percent of students chose to make their attendance mandatory.

#### Attendance outcomes

We analyzed 794 observations using a logistic mixed-effects model. Our dependent variable was class attendance (attended versus absent). We included fixed effects for attendance policy, meeting number, and TA, along with interactions between these variables. To account for the repeated-measures design, we included a random intercept, which improved the model’s fit substantially: marginal *R^2^* = 0.08, conditional *R^2^* = 0.31, Likelihood Ratio Test (LRT) χ*^2^*(12) = 105, *P* < 0.001. Around 25% of the variance in attendance was attributable to differences between students (see the Supplementary Materials for more details and for alternative model specifications).

**Fig. 1.**
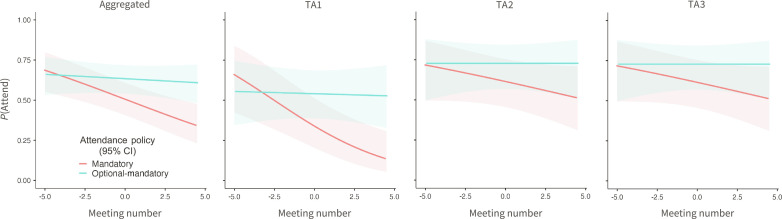
Students given greater autonomy attended class more reliably. The probability of students attending class by week, overall (left) and disaggregated by teaching assistant (TA1 to TA3). Confidence intervals are 95%. Students in the mandatory groups—who were not allowed to choose whether to make attendance count toward their grades—became less likely to attend class as the semester progressed. But students in the optional-mandatory groups—90% of whom had chosen at the beginning of semester to make their attendance count toward their grades—maintained high attendance rates across the semester.

Compared to students in the mandatory groups, optional-mandatory students were more likely to attend weekly sections [odds ratio (OR) = 1.7, 95% CI 0.96 to 2.9], but this effect did not meet the conventional standard of statistical significance: *P* = 0.072. However, while all students were relatively motivated at the start of the semester—nearly 75% attended the first meeting of their section—we found a significant interaction between policy and meeting number (i.e., week): OR = 1.14, 95% CI 1.0 to 1.3, *P* = 0.025. Simple effects tests revealed that attendance in the mandatory groups declined over the course of the semester: χ*^2^* = 13, OR = 0.86, 95% CI 0.79 to 0.93, *z* = −3.6, *P* < 0.001. However, it was stable in the optional-mandatory groups: χ^2^ = 0.37, OR = 0.98, 95% CI 0.91 to 1.1, *z* = −0.61, *P* = 0.541 (Fig. 1). The pattern emerged across all sections, and we observed no interaction between policy and TA (all *P*s ≥ 0.397). These results were robust across alternative model specifications (see “Alternative Models” in the Supplementary Materials).

Despite the high student uptake of mandatory attendance, the minority of students who choose optional attendance could be cause for some concern. Will these students fall through the cracks? To preserve students’ perceptions of autonomy, we did not require those who opted for fully optional attendance to register their presence in class when they chose to attend. For this reason, we cannot say how these students behaved. However, while we acknowledge that it is possible they opted out of attending class altogether, it is worth noting that choosing optional attendance is not the same as not attending; it merely implies maintaining flexibility regarding attendance. Even for students who opt out, optional-mandatory may discourage truancy because it fosters intrinsic motivation. Additionally, it is worth remembering that mandatory attendance policies do not ensure perfect attendance. Students still miss class; they just take penalties for doing so.

### Study 2: Assessment options

Regular assessment followed by timely feedback is a crucial ingredient of effective teaching (*36*, *37*). However, students often seek to minimize the effort required to complete assignments (*38*, *39*). Teachers can push students to spend more time and effort on their assignments by weighting them more heavily, but such strategies can backfire by causing stress that may hurt both academic performance and well-being [e.g., (*40*)]. The challenge, then, is to induce students to work hard without the use of controlling incentives.

One approach is to give students greater control over how they are assessed. Forcing students to complete assignments can lead to reactance due to a loss of autonomy (*41*, *42*). This can result in students devoting less time and effort to the assignments, procrastinating, or foregoing the assignment entirely. Giving students a choice of assessment options might allow for a greater sense of autonomy, which as we have seen can powerfully bolster motivation.

To test this hypothesis, we ran a study on students in an introductory philosophy course taught over two fall semesters by the same professor at the same institution (*n* = 114). We assigned students to treatment groups by cohort:

1) In the first cohort (“mandatory”), all students were required to complete a series of 20 challenging argument analysis problem sets (*m*_difficulty_ = 3.8/5, 95% CI 3.7 to 3.8, where “5” = “Extremely difficult”). Problem sets were designed to train high-level reasoning skills using a technique known as “argument visualization” (*43*).

2) In the second cohort (“free-to-switch”), students chose between problem set–based assessment and a second option—essay-based assessment—which required relatively little work. Students who chose this second option were assessed based on weekly reading questions and short (five-page) midterm and final essays. Students were made aware of the relative difficulty of the two options and were allowed to switch into the low-effort, essay-based assessment stream at any point before the midterm essay deadline.

Because our study assigned students to treatment groups in cohorts, we cannot rule out the possibility of unmeasured confounds. However, it is reassuring to note that there were no meaningful differences in students’ seniority, home college, home department, or major (all *P*s ≥ 0.392, γ = −0.030 to −0.040; see the Supplementary Materials for detailed comparisons).

Ninety percent of students in the free-to-switch cohort initially set out to pursue problem set–based assessment, and only 5% of those students later switched into the essay-based stream. If the students who chose the low-effort assessment option were weaker than average, including their data could increase the relative performance of the free-to-switch cohort. We therefore excluded submissions from students ranked below the 15th percentile in the mandatory cohort. Although this represents a conservative assumption that only the lowest-performing students opted out, it did not affect our results. To ensure our comparison was based solely on students completing assignments with identical instructions, we excluded assignments that changed between cohorts. After these exclusions, 1036 submissions remained for analysis. In this study, we explored two dependent variables. First, students’ self-reported time on each problem set and second, the quality of their work as assessed by TAs blind to the hypothesis under study.

#### Problem set effort

Whenever students submitted problem sets, they reported how long they spent on the homework assignment using a six-point scale. We constructed an ordinal logistic mixed model with fixed effects for assessment policy (mandatory versus free-to-switch), problem set number (1 to 12), their interaction, and a random intercept to account for the repeated-measures design. The inclusion of the random intercept significantly improved the model fit: marginal *R^2^* = 0.080, conditional *R^2^* = 0.44, LRT χ*^2^*(4) = 342, *P* < 0.001. Approximately 39% of the total variance in time spent on assignments could be attributed to differences between students.

**Fig. 2.**
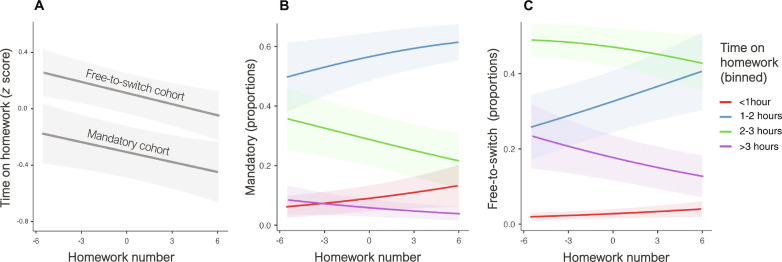
Students given greater autonomy invested more effort into their work. (**A**) Students in the mandatory cohort were forced to complete a demanding series of problem sets. In the free-to-switch cohort, students were allowed to switch to a less demanding assessment option at any point before the midterm essay deadline. All students spent less time on problem sets as the semester progressed. However, students in the free-to-switch cohort, who chose to complete the problem sets, consistently worked harder than students in the mandatory cohort, who were forced to complete the same assignments. (**B**) Probability of a submission being in each time category in the mandatory cohort: As the semester progressed, these students became far less likely to report spending 2 to 3 hours on their assignments and more likely to report spending less than 1 hour. (**C**) By contrast, students in the free-to-switch cohort were far more likely to report spending 2 to 3 hours and >3 hours than were students in the mandatory cohort.

Across both cohorts, each additional assignment reduced the odds of reporting a higher time investment by around 7%: OR = 0.93, 95% CI 0.90 to 0.97, *z* = −3.8, *P* < 0.001. We found no interaction between assignment number and cohort, indicating that the trend of spending less time on homework assignments as the semester progressed was consistent across the groups: χ*^2^*(1) = 0.0060, *P* = 0.937. Crucially, a significant effect of cohort emerged: χ*^2^*(1) = 15, *P* < 0.001. Relative to students in the mandatory cohort, students in the free-to-switch cohort had 3.6 times the odds of reporting a higher category of time invested on homework assignments: 95% CI 1.9 to 6.9, *z* = 3.1, *P* = 0.002 (Fig. 2). As in Study 1, our qualitative conclusions are robust to alternative model specifications.

#### Problem set performance

Problem sets were graded on a three-point scale (0, 1, 2). The standard for earning one point was submitting a “meaningful attempt” before the deadline. After excluding the seven lowest-performing students in the mandatory cohort (as explained above), all submissions received either a “1” or “2.” In the free-to-switch cohort, only 0.16% of submissions received a “0.” For ease of interpretation, we excluded these submissions and tested a logistic mixed-effects model. This decision did not change the qualitative pattern of results (see the Supplementary Materials for alternative models and model specifications).

We fitted a logistic mixed-effects model using a logit link function and a binomial distribution. The model included fixed effects for assessment policy (mandatory versus free-to-switch), homework assignment number (1 to 12), and their interaction, and a random intercept to account for the repeated-measures design. The inclusion of the random intercept significantly improved the model fit: marginal *R^2^* = 0.10, conditional *R^2^* = 0.24, LRT χ*^2^*(4) = 87, *P* < 0.001.

**Fig. 3.**
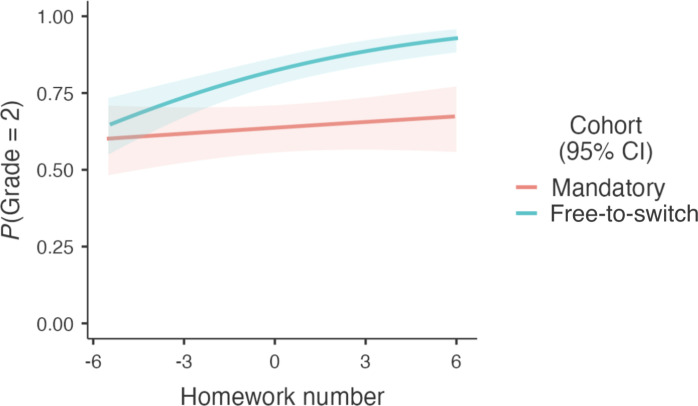
Students given greater autonomy attained greater proficiency with course materials. Over the course of the semester, students in the free-to-switch cohort became more likely to earn a “2” on the problem sets. Students in the mandatory cohort did not improve significantly. The difference between the two cohorts’ trajectories may be partly explained by optional-mandatory students spending more time on their homework assignments (Fig. 2).

We predicted that higher motivation in the free-to-switch cohort would, over time, translate into greater proficiency as manifested in students’ grades. Confirming this prediction, a statistically robust interaction between assessment policy and assignment number emerged: OR = 1.16, 95% CI 1.06 to 1.26, *z* = 3.2, *P* = 0.002. For each additional assignment, the odds of achieving a grade of “2” increased by roughly 16% more in the free-to-switch cohort than in the mandatory cohort (Fig. 3). Simple effects tests revealed that while students did not improve significantly when they were forced to complete problem sets (χ^2^ = 0.76, *P* = 0.383), they did improve when they were given greater autonomy (χ^2^ = 28, *P* < 0.001). As before, this pattern emerged across alternative model specifications.

This cohort study illustrates several points:

1) Eighty-five percent of students opted for a more demanding assessment option and stuck with their choice. In two subsequent classes, 94 to 97% of students did the same (*N*s = 157 and 197). Direct comparisons between these classes and the cohorts studied here are not possible due to differences in course materials. However, we have consistently replicated the widespread student adoption of a high-effort assessment option.

2) Giving students meaningful control over their own learning increased the time they spent studying and improved their performance on complex, high-level reasoning tasks.

3) Although few students in the free-to-switch cohort chose the less demanding essay-based assessment, the mere availability of this choice appeared to boost motivation and learning.

This suggests that controlling mandates are not always needed to engage students in rigorous learning, and that increasing student autonomy can provide powerful motivational benefits even in the absence of a binding pre-commitment.

It is important to acknowledge that some students ultimately chose the less challenging option, which may have resulted in a less rigorous learning experience compared to those who opted for the more demanding alternative. However, it is not clear that those students would have engaged more meaningfully with the material even if problem sets had been mandated—it is not uncommon for students to fail to turn in required assignments and simply take the penalty to their grades.

## DISCUSSION

Many resources are available for educators to improve students’ perceptions of belongingness and mastery. However, there is a dearth of research validating interventions to promote autonomy in higher education. This is despite a large literature consistently showing the importance of autonomy in driving intrinsic motivation in general (*4*, *5*) and in the classroom (*15*, *44*). Indeed, in a recent large-scale review of choice architecture interventions, fewer than 10 targeted education and none were related to promoting autonomy (*45*). Our evaluation of resources from over a dozen prominent university teaching and learning centers revealed almost no advice for implementing autonomy-supportive policies in undergraduate classrooms (Table 1). By addressing this gap, these centers can better assist instructors in implementing evidence-based practices that foster motivation, proficiency, and well-being. This is particularly important in STEM fields where there is considerable interest in improving persistence rates [e.g., (*46*, *47*)].

To begin addressing this lack of autonomy-supportive interventions in higher education, we developed and investigated two classroom interventions that can be implemented with minimal financial or logistical costs, and we provided empirical evidence for their effectiveness. Allowing students to commit to mandatory attendance and to choose more rigorous assignments led them to attend class more reliably, to put more effort into their assignments, and to understand the material better. While we have focused on attendance and assessment, similar choice architectures can be applied to many other course elements. For any mandatory course element, it is worth considering whether making it optional-mandatory might serve students better (Table 2).

**Table 2.** Examples of autonomy-enhancing course policies. These interventions share two key features: They give students greater control over their learning, and they are experimentally testable.

Intervention	Description
Deadlines	Instead of purely faculty-set deadlines, students could have a say in choosing their own, allowing for greater autonomy and better schedule management [see (*81*)].
Extensions	Students could be given a number of “extension days” to use at their discretion over the semester, removing subjectivity from the extension process while boosting student motivation.
Materials	Rather than dictating course materials, instructors could offer multiple options and formats for readings and provide supplemental resources for students who want to deepen their understanding.
Exams and projects	Giving students a selection of exam questions or project formats can also enhance autonomy. Students could select which questions to answer or choose their final project medium, subject to instructor approval [see (*82*)].
Assessment weights	Permitting students to allocate weight to different course elements within set bounds could enhance their sense of autonomy and motivation.
Supplementary topics	While core topics are nonnegotiable, supplementary topics could be chosen by student vote, increasing engagement by aligning course material with student interest.

Despite these promising initial results, several caveats are in order when interpreting our data. First, our data represent students from just one school. Carnegie Mellon is a selective university known for attracting students with a strong work ethic. Our studies did not measure participants’ demographic characteristics, and therefore cannot speak to how such characteristics may interact with autonomy-supportive policies. Thus, it is possible that these interventions affect different populations differently. However, academically at-risk students also experience educational benefits from autonomy-supportive learning environments (*48*).

Carnegie Mellon is a culturally diverse institution, ranking 14th out of over 1200 colleges and universities in international popularity, with nearly one in six students coming from outside the United States (*49*). Nevertheless, the university is situated within a Western individualist cultural context, and our results may not generalize to other contexts. However, the benefits of autonomy-supportive interventions have been demonstrated across multiple cultures (*50*, *51*).

All our participants were college-age students. While we expect our results to generalize to older students, it is less certain how they translate to younger demographics, such as elementary school students. However, decades of research amply demonstrate the importance of autonomy for children of all ages (*52*). Although the specific interventions explored here may not directly transfer to younger students, the underlying principle of fostering autonomy remains crucial (*53*). Moreover, there were undoubtedly neurodiverse individuals in our samples. However, we cannot isolate the policies’ effect on them. Some evidence suggests that autonomy-supportive policies are especially important for neurodiverse individuals (*54*–*57*). While this work is certainly encouraging, it cannot license a confident generalization of the present results to these populations.

The present studies were run exclusively on popular introductory level courses in the humanities and social sciences. While we are not aware of any reason to suspect that the material covered was especially conducive to demonstrating the benefits of autonomy, it remains an empirical question whether our findings extend to more technical material. Similarly, autonomy promotion may have a different effect when applied to electives as opposed to required courses, which by their very nature restrict autonomy. The extent to which autonomy-promoting interventions interact with other features of a class—such as whether it is perceived to be valuable, whether students have intrinsic interest in the material, and the quality of teaching—also remains unclear.

In short, research should explore the generalizability of our results across multiple dimensions. These include the selectivity of the educational institution, the cultural context in which the institution is situated, the age and neurodiversity of the participants, and the nature of the course content and delivery modality. In future studies, we plan to investigate how these factors interact with autonomy-supportive course policies, thereby providing a more comprehensive understanding of their effectiveness and boundary conditions.

Issues of generalization aside, several weaknesses in the present studies should be acknowledged. First, sample sizes were limited by the number of students enrolled in the courses where the interventions were tested. Moreover, university and institutional review board (IRB) policies limited our ability to randomly assign participants to treatment groups. For instance, in Study 1, discussion sections were randomly assigned to implement either a mandatory or optional-mandatory attendance policy. However, following standard university procedures, students were not randomly assigned to sections. This lack of random assignment might have introduced various confounds, dependencies, or failures of randomization. For example, if groups of students from a particular extracurricular activity all enrolled in the same section, it could have led to nonrandom clustering of participants. Similarly, in Study 2, different policies were adopted in different semesters, as it is not feasible to adopt different grading standards for students in the same class during the same semester. It is possible that there were unmeasured differences in the composition of the cohorts or subtle differences in how the class was taught that were unrelated to the intervention, but which influenced the results. As is often the case with field studies, the trade-off for increased naturalistic validity is a loss of some experimental control.

Finally, our studies do not offer much mechanistic insight. The choice architecture we applied yielded meaningful improvements in both subjective and objective measures, but we lack evidence to make strong claims about why the interventions were so successful.

It is clear that increasing autonomy per se is not sufficient to yield results like those reported here. After all, making attendance fully optional preserves student autonomy but results in lower turnout [e.g., (*27*, *28*)]. Why, then, was optional-mandatory attendance so successful? One reason may be due to when the choices are made. In an optional-mandatory regime, students opt into mandatory attendance exactly once: at the beginning of the term, before they are overwhelmed by a heavy workload and when their identity as a scholar is most psychologically salient (*58*). In a purely optional regime, students are forced to make difficult trade-offs repeatedly over the course of the semester (“Do I trudge through the snow to get to class or stay home in comfort?”). The more often students face such decisions, the more likely they are to sometimes choose not to come to class. By having students pre-commit to attendance when these trade-offs are not as salient, the choice architecture provides choice in a context when students are most likely to make educationally beneficial decisions. Of course, this is speculative, and future research should explore such mechanisms in greater detail.

While the effectiveness of pre-commitment strategies has been established in other contexts—e.g., health (*59*) and gambling addiction (*60*)—there is little work on the subject in higher education. The results of Study 1 suggest that pre-commitment is an important tool in the choice architects’ toolbox for higher education, and future research should explore its implications more thoroughly. However, it is also important to note that our findings cannot be entirely explained by the effectiveness of pre-commitment. In Study 2, students in the free-to-switch cohort showed greater engagement and improvement than those in the mandatory cohort, despite having the option to abandon their initial choice at any time before the midterm essay deadline. This suggests that autonomy itself, even in the absence of strong pre-commitment, can be a powerful motivator.

Besides educational gains, fostering student autonomy can yield broader benefits. A sense of personal autonomy is linked to both better physical (*61*–*63*) and mental health (*64*, *65*). This is crucial given the high rates of depression and anxiety observed among college students (*66*, *67*). Moreover, giving students meaningful control over their own learning, even if they occasionally err, serves as practical training for decision-making. Studies suggest that an error rate of about 15% is optimal for learning across many domains (*68*). If we aim to promote independent decision making, incorporating autonomy in the classroom can have long-lasting advantages.

Finally, students juggle various responsibilities beyond academics, balancing careers, caregiver duties, service activities, health care needs, and so on. Although they may be well-intentioned, faculty-imposed mandates can place an undue burden on students, particularly those who are disadvantaged and/or disabled. Autonomy-supportive policies empower students to better manage their complex lives.

Evidence for the importance of student autonomy has been accumulating for decades (*4*), so why has it had so little impact on higher education? One possibility is that faculty doubt that students will make good decisions [see (*24*)]. When we informally polled our faculty colleagues on their expectations for the present studies, most believed that fewer than 20% of students would opt into mandatory attendance (whereas 73 to 95% actually do). They also believed that almost no students would voluntarily complete problem sets (whereas 80 to 97% actually do). Why are faculty predictions about student decisions so inaccurate?

One explanation is that faculty are too quick to explain students’ behavior in terms of stable dispositions rather than environmental factors [(*69*); but see (*70*)]. Students do care about their academic success and consider their scholar-self as a core part of their identity (*58*). However, when situational constraints, such as coming to campus during a blizzard, difficulty finding a parking space, needing to study for another exam, or a personal crisis, prevent students from attending class, professors may mistakenly attribute the absence to the student’s personality (e.g., “Students just don’t care about my class like they used to”) rather than the situational constraints.

Faulty intuitions about the drivers of human motivation might also make faculty reluctant to grant students greater autonomy. For example, although people believe that they are more likely to persist in challenging behaviors such as dieting and exercise when they focus on long-term benefits, research suggests that attending to immediate rewards leads to greater persistence (*71*). Faculty might similarly turn to autonomy-undermining extrinsic motivators if they forget that learning should be inherently fun and rewarding. Moreover, research on preferences for paternalistic policies in educational contexts reveals that a meaningful fraction of people believe it is a professor’s job to protect students from making poor decisions in and even outside of the classroom (*72*). Thus, paternalistic attitudes could lead faculty to believe that they must make wise decisions on students’ behalf.

Of course, another possibility is that faculty want to give students more autonomy but lack tools for doing so. We hope that this article will provide them with ideas while galvanizing further research on practical, autonomy-promoting classroom interventions.

We have focused here on interventions to increase student autonomy, but it is worth mentioning that research has found that members of the teaching team also benefit from autonomy-supportive policies (*73*). TAs are often given little choice over what their sections will focus on, what their schedule will look like, what assignments they will grade, or when they will grade them. While faculty are typically afforded much more autonomy in how they teach, there is a growing tendency to impose top-down requirements on professors. For example, some schools standardize content across sections, have requirements about language that must be included in syllabi, and restrict what classes faculty can teach. Because autonomy is likely to be as motivating to teachers as it is to students, administrators would be wise to consider how campus policies could be changed to promote teachers’ autonomy.

The role of autonomy promotion outside of specific classrooms and throughout the wider curriculum is also worth considering. Required classes, whether they be for general education or toward completing a major or degree program, by their nature restrict choice. To the extent that students perceive a curricular requirement as threatening their autonomy, they may experience psychological reactance (*74*), which can undermine whatever intrinsic interest they would otherwise have in the topic (*75*). Exposure to autonomy-restricting policies throughout schooling has been repeatedly linked to decreased love of learning [e.g., (*76*)].

The shift to online and hybrid education presents both opportunities and risks for student autonomy. Completion and retention rates of massive open online courses (MOOCs) and other online courses remain disturbingly low—often below 15% (*77*, *78*). Promoting autonomy in online education might improve persistence rates. However, online students face countless distractions, some of which may prove too tempting to resist [(*79*); however, see (*80*) for a promising example]. Similarly, new technologies can be used to support autonomy or to restrict it. This is an area ripe for exploration by choice architects.

Much research in psychology has demonstrated that autonomy is a core human need and a fundamental component of intrinsic motivation (*4*, *5*). However, despite good evidence that this applies in educational settings, there are surprisingly few studies that explore concrete interventions for increasing student autonomy in higher education. University teaching and learning centers and many other resources intended to help educators improve their craft largely neglect autonomy support. Here, we have identified two interventions and provided evidence for their effectiveness in higher education. We have also suggested other places in the classroom and curriculum where autonomy-supportive approaches might be profitably applied. We leave the choice of whether to apply them in your own classroom up to you.

## METHODS

### Student survey

Participants were 101 students who had taken at least one course with at least one of the authors. After providing informed consent, participants were asked about their experiences with seven different course policies, including mandatory attendance (“Your attendance is tracked, and you are penalized if you are absent”), optional attendance (“There is no record of whether or not you attend class, and attending is not obligatory”), and optional-mandatory attendance (“By default, there is no attendance record and you are not required to attend. However, you may choose to sign up for mandatory attendance; if you do, your attendance will be tracked and you will be penalized if you are absent”). Participants indicated whether they had experience with each policy, and if so, whether it had been in the context of a required or elective class. Participants then used 10-point scales to indicate how they believed the policy affected their motivation, enjoyment, learning, relationship with the teaching team, and their likelihood of taking another course with the policy. Students who indicated no experience with a policy were asked to predict how it would affect them. Survey questions are reproduced in the Supplementary Materials, and survey data can be found in this article’s Dryad repository.

#### Study 1

Participants were 104 students enrolled in a large Gen-Ed philosophy course at Carnegie Mellon University. Discussion sections were taught by TAs blind to the hypothesis under study. TAs were randomly assigned to teach one section under a mandatory attendance policy and one under an optional-mandatory policy. Sections met either at 1 p.m. or 2 p.m. on most Fridays of the semester. To reduce the probability of a conflict with another course or extracurricular biasing our results, Mandatory and Optional-Mandatory sections were counterbalanced across meeting times. One TA lost their attendance records, and we therefore base our analysis on the six sections for which we have complete data.

In the first meeting of the semester, students in the mandatory sections were told by their TAs: “In this class, you are permitted to miss up to three recitations without permission or penalty. If you miss no more than three recitations, you will receive +3% on your final grade. If you do not keep your commitment by missing three or more recitations, your final score will be docked by −3%.”

Students in the optional-mandatory sections were told: “In this class, you choose if you would like your attendance at recitations to count toward your final grade. Students who choose to make attendance count will be assessed as follows. You are permitted to miss up to three recitation meetings without permission or penalty. If you miss no more than three meetings, you will receive +3% on your final grade. If you miss three or more recitations, your final grade will be docked by −3%. If you choose not to make your attendance count, whether you attend will not affect your grade.”

Thus, when students in the optional-mandatory condition chose to make their attendance mandatory, they exposed themselves to the same policy as students in the mandatory condition. Students used a custom mobile app to participate in class discussions. Anonymized records from the app were used to generate attendance reports for 10 section meetings distributed across the semester.

#### Study 2

Participants were 114 students in an introductory philosophy course offered in two fall semesters by the same professor at the same institution:

1) In the first cohort, Mandatory (M), all students were required to complete problem sets.

2) In the second cohort, Free-to-Switch (F), students were given the same choice and were also allowed to switch out of problem set–based assessment and into the essay-based stream at any time before the deadline for the first essay (midterm).

Across both cohorts, students submitted 1693 problem sets. To maximize comparability between cohorts, we included only homework assignments that were offered without modification across cohorts. This excluded 304 submissions from M and 246 from F. Submissions from students who dropped the course were excluded, removing 28 submissions (six students) in M and 13 (nine students) in F. Only 11 students in F, representing 15% of their cohort, chose the lower-effort essay-based assessment option. Consequently, to control for potential advantages in this cohort, we excluded the seven students in M ranked below the 15th percentile of their cohort (41 submissions). Our final dataset consisted of a total of 1036 submissions representing 98 students.

Problem sets required students to analyze argumentative philosophical texts by creating argument visualizations—diagrams designed to lay bare the inferential structures implicit in argumentative prose (*43*). When students submitted a problem set for assessment, they reported how difficult they found it and how long they worked on it using a six-point scale: “Less than 1 hour,” “1 to 2 hours,” “2 to 3 hours,” “3 to 4 hours,” “4 to 5 hours,” and “More than 5 hours.” In 39% of submissions, students reported spending between 1 and 2 hours on each homework assignment, and a further 52% reported spending over 2 hours.

Because study 2 assigned students to treatment groups in cohorts, we cannot rule out the possibility of unmeasured confounds. However, it is reassuring to note that there were no meaningful differences between cohorts in students’ seniority, χ*^2^*(3) = 1.7, *P* = 0.627, γ = −0.030, home department, χ*^2^*(23) = 20, *P* = 0.639, γ = −0.040, or major, χ*^2^*(26) = 27, *P* = 0.392, γ = −0.035. We report values for both chi-squared and gamma to provide a comprehensive view of the associations; the former assesses the significance of the associations, while the latter quantifies their strength and direction in a nonparametric manner.

A third cohort was also tested; however, due to unforeseen staffing changes, many problem sets in this cohort remained ungraded or were graded by the instructor who was not blind to the hypothesis under study. Despite these limitations, the overall pattern of results aligned with our theoretical account. Descriptive statistics for the third cohort can be found in the Supplementary Materials, and all data can be found in this article’s Dryad repository.

## Supplementary Materials

### This PDF file includes:

Figs. S1 to S7 Tables S1 to S3 Alternative Models
