# Supplementary material for: Choosing to learn: The importance of student autonomy in higher education
Source: Sci Adv. 2024 Jul 17;10(29):eado6759. doi: 10.1126/sciadv.ado6759 (PMC466944; doi:10.1126/sciadv.ado6759)
Supplement: Supplementary file 1 — Figs. S1 to S7 Tables S1 to S3 Alternative Models [file sciadv.ado6759_sm.pdf]

Science Advances
AAAS

# Supplementary Materials for

## Choosing to learn: The importance of student autonomy in higher education

Simon Cullen and Daniel Oppenheimer

Corresponding author: Simon Cullen, cullen@cmu.edu.

*Sci. Adv.* **10**, eado6759 (2024)
DOI: 10.1126/sciadv.ado6759

**This PDF file includes:**

Figs. S1 to S7
Tables S1 to S3
Alternative Models

**Table S1.**

Comparison of student evaluations for mandatory and optional attendance policies.

Policy descriptions:

1. Mandatory Attendance: Your attendance is tracked, and you are penalized if you are absent
2. Optional Attendance: There is no record of whether or not you attend class, and it is not obligatory.
3. Optional-Mandatory Attendance: As a default, there is no record to attend class and it is not obligatory. However, you may choose to sign up for mandatory attendance; if you do your attendance will be tracked and you are penalized if you are absent. However, by opting into mandatory attendance you will guarantee that all your teammates for group projects have also opted into mandatory attendance.

Independent Samples T-Test

| | | | | | | | 95% Confidence Interval | | | | 95% Confidence Interval | |
|---|---|---|---|---|---|---|---|---|---|---|---|---|
| | | Statistic | df | p | Mean difference | SE difference | Lower | Upper | | Effect Size | Lower | Upper |
| Enjoyment | Student's t | 5.101 | 111.000 | < .001 | 1.916 | 0.376 | 1.172 | 2.660 | Cohen's d | 0.962 | 0.569 | 1.350 |
| | Mann-Whitney U | 752.500 | | < .001 | 2.000 | | 1.000 | 2.000 | Rank biserial correlation | 0.527 | | |
| Motivation | Student's t | −0.992 | 111.000 | 0.324 | −0.452 | 0.456 | −1.355 | 0.451 | Cohen's d | −0.187 | −0.557 | 0.184 |
| | Mann-Whitney U | 1387.000 | | 0.236 | −0.000 | | −1.000 | 0.000 | Rank biserial correlation | 0.128 | | |
| Learning | Student's t | −2.359 | 111.000 | 0.020 | −0.920 | 0.390 | −1.693 | −0.147 | Cohen's d | −0.445 | −0.818 | −0.070 |
| | Mann-Whitney U | 1172.500 | | 0.013 | −1.000 | | −1.000 | −0.000 | Rank biserial correlation | 0.263 | | |
| Relationship | Student's t | −0.245 | 111.000 | 0.807 | −0.111 | 0.451 | −1.005 | 0.784 | Cohen's d | −0.046 | −0.416 | 0.323 |
| | Mann-Whitney U | 1541.500 | | 0.775 | 0.000 | | −0.000 | 1.000 | Rank biserial correlation | 0.031 | | |
| Likelihood | Student's t | 6.353 | 111.000 | < .001 | 2.226 | 0.350 | 1.532 | 2.920 | Cohen's d | 1.198 | 0.794 | 1.597 |
| | Mann-Whitney U | 603.000 | | < .001 | 2.000 | | 1.000 | 3.000 | Rank biserial correlation | 0.621 | | |
| OverallEvaluation | Student's t | 3.208 | 111.000 | 0.002 | 1.343 | 0.419 | 0.514 | 2.173 | Cohen's d | 0.605 | 0.226 | 0.981 |
| | Mann-Whitney U | 971.000 | | < .001 | 1.000 | | 1.000 | 2.000 | Rank biserial correlation | 0.389 | | |
| PeerEvaluation | Student's t | 8.478 | 111.000 | < .001 | 3.602 | 0.425 | 2.760 | 4.444 | Cohen's d | 1.598 | 1.171 | 2.020 |
| | Mann-Whitney U | 427.000 | | < .001 | 4.000 | | 3.000 | 5.000 | Rank biserial correlation | 0.731 | | |

*Note.* $H_a$ $\mu_{Optional} \neq \mu_{Mandatory}$

**Table S2**

Comparison of student evaluations for optional and optional-mandatory attendance policies

Independent Samples T-Test

| | | Statistic | df | p | Mean difference | SE difference | 95% Confidence Interval Lower | 95% Confidence Interval Upper | | Effect Size | 95% Confidence Interval Lower | 95% Confidence Interval Upper |
|---|---|---|---|---|---|---|---|---|---|---|---|---|
| Enjoyment | Student's t | −1.982 | 110.000 | 0.050 | −0.701 | 0.354 | −1.403 | 0.000 | Cohen's d | −0.375 | −0.749 | 0.000 |
| | Mann-Whitney U | 1202.500 | | 0.031 | −1.000 | | −1.000 | −0.000 | Rank biserial correlation | 0.229 | | |
| Motivation | Student's t | 2.355 | 110.000 | 0.020 | 1.015 | 0.431 | 0.161 | 1.870 | Cohen's d | 0.446 | 0.069 | 0.821 |
| | Mann-Whitney U | 1130.500 | | 0.011 | 1.000 | | 0.000 | 2.000 | Rank biserial correlation | 0.275 | | |
| Learning | Student's t | 2.882 | 110.000 | 0.005 | 1.072 | 0.372 | 0.335 | 1.809 | Cohen's d | 0.546 | 0.167 | 0.923 |
| | Mann-Whitney U | 1036.500 | | 0.002 | 1.000 | | 0.000 | 2.000 | Rank biserial correlation | 0.336 | | |
| Relationship | Student's t | 0.991 [a] | 110.000 | 0.324 | 0.406 | 0.410 | −0.406 | 1.219 | Cohen's d | 0.188 | −0.185 | 0.560 |
| | Mann-Whitney U | 1415.500 | | 0.378 | 0.000 | | −0.000 | 1.000 | Rank biserial correlation | 0.093 | | |
| Likelihood | Student's t | −3.113 [a] | 109.000 | 0.002 | −1.002 | 0.322 | −1.640 | −0.364 | Cohen's d | −0.593 | −0.973 | −0.210 |
| | Mann-Whitney U | 1037.000 | | 0.002 | −1.000 | | −2.000 | −0.000 | Rank biserial correlation | 0.322 | | |
| OverallEvaluation | Student's t | 0.413 | 110.000 | 0.681 | 0.172 | 0.416 | −0.653 | 0.997 | Cohen's d | 0.078 | −0.293 | 0.450 |
| | Mann-Whitney U | 1544.500 | | 0.929 | 0.000 | | −1.000 | 1.000 | Rank biserial correlation | 0.010 | | |
| PeerEvaluation | Student's t | −3.025 | 110.000 | 0.003 | −1.185 | 0.392 | −1.961 | −0.409 | Cohen's d | −0.573 | −0.951 | −0.193 |
| | Mann-Whitney U | 1022.500 | | 0.001 | −1.000 | | −2.000 | −0.000 | Rank biserial correlation | 0.345 | | |

*Note.* $H_a$ $\mu_{OptionalMandatory} \neq \mu_{Optional}$

[a] Levene's test is significant ($p < .05$), suggesting a violation of the assumption of equal variances

**Table S3**

Comparison of student evaluations for mandatory and optional-mandatory attendance policies

Independent Samples T-Test

| | | Statistic | df | p | Mean difference | SE difference | 95% Confidence Interval Lower | 95% Confidence Interval Upper | | Effect Size | 95% Confidence Interval Lower | 95% Confidence Interval Upper |
|---|---|---|---|---|---|---|---|---|---|---|---|---|
| Enjoyment | Student's t | 3.108 | 103.000 | 0.002 | 1.214 | 0.391 | 0.439 | 1.989 | Cohen's d | 0.607 | 0.214 | 0.997 |
| | Mann-Whitney U | 890.500 | | 0.001 | 1.000 | | 0.000 | 2.000 | Rank biserial correlation | 0.354 | | |
| Motivation | Student's t | 1.217 | 103.000 | 0.226 | 0.563 | 0.463 | −0.354 | 1.481 | Cohen's d | 0.238 | −0.147 | 0.621 |
| | Mann-Whitney U | 1175.000 | | 0.187 | 0.000 | | −0.000 | 1.000 | Rank biserial correlation | 0.147 | | |
| Learning | Student's t | 0.414 | 103.000 | 0.680 | 0.152 | 0.367 | −0.575 | 0.879 | Cohen's d | 0.081 | −0.302 | 0.463 |
| | Mann-Whitney U | 1273.000 | | 0.484 | 0.000 | | −0.000 | 1.000 | Rank biserial correlation | 0.076 | | |
| Relationship | Student's t | 0.777 | 103.000 | 0.439 | 0.296 | 0.381 | −0.459 | 1.051 | Cohen's d | 0.152 | −0.232 | 0.534 |
| | Mann-Whitney U | 1127.000 | | 0.088 | 0.000 | | −0.000 | 1.000 | Rank biserial correlation | 0.182 | | |
| Likelihood | Student's t | 3.929 | 102.000 | < .001 | 1.224 | 0.311 | 0.606 | 1.842 | Cohen's d | 0.771 | 0.370 | 1.168 |
| | Mann-Whitney U | 753.500 | | < .001 | 1.000 | | 0.000 | 1.000 | Rank biserial correlation | 0.442 | | |
| OverallEvaluation | Student's t | 3.254 | 103.000 | 0.002 | 1.515 | 0.466 | 0.592 | 2.438 | Cohen's d | 0.635 | 0.241 | 1.026 |
| | Mann-Whitney U | 867.000 | | < .001 | 1.000 | | 1.000 | 2.000 | Rank biserial correlation | 0.371 | | |
| PeerEvaluation | Student's t | 5.530 | 103.000 | < .001 | 2.417 | 0.437 | 1.550 | 3.284 | Cohen's d | 1.079 | 0.667 | 1.487 |
| | Mann-Whitney U | 554.000 | | < .001 | 3.000 | | 2.000 | 3.000 | Rank biserial correlation | 0.598 | | |

*Note.* $H_a$ $\mu_{OptionalMandatory} \neq \mu_{Mandatory}$

**Figure S1**

How did [POLICY] affect your enjoyment of the class?

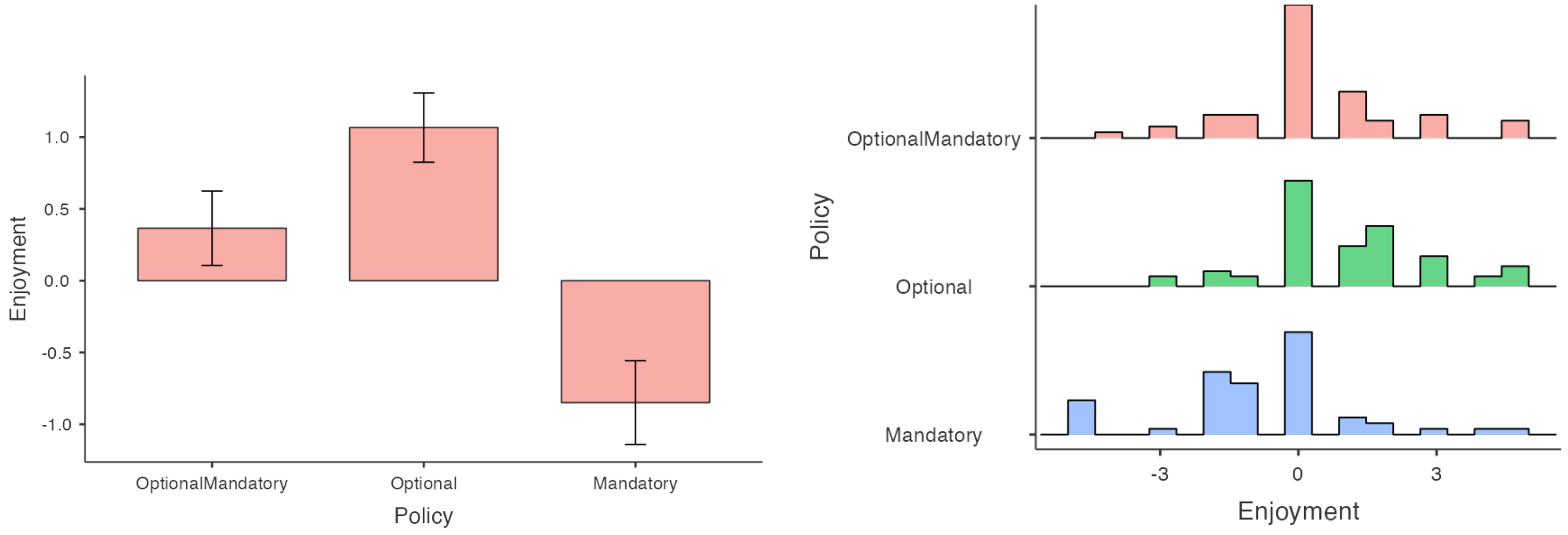

**Figure S2**

How did [POLICY] affect your motivation in the class?

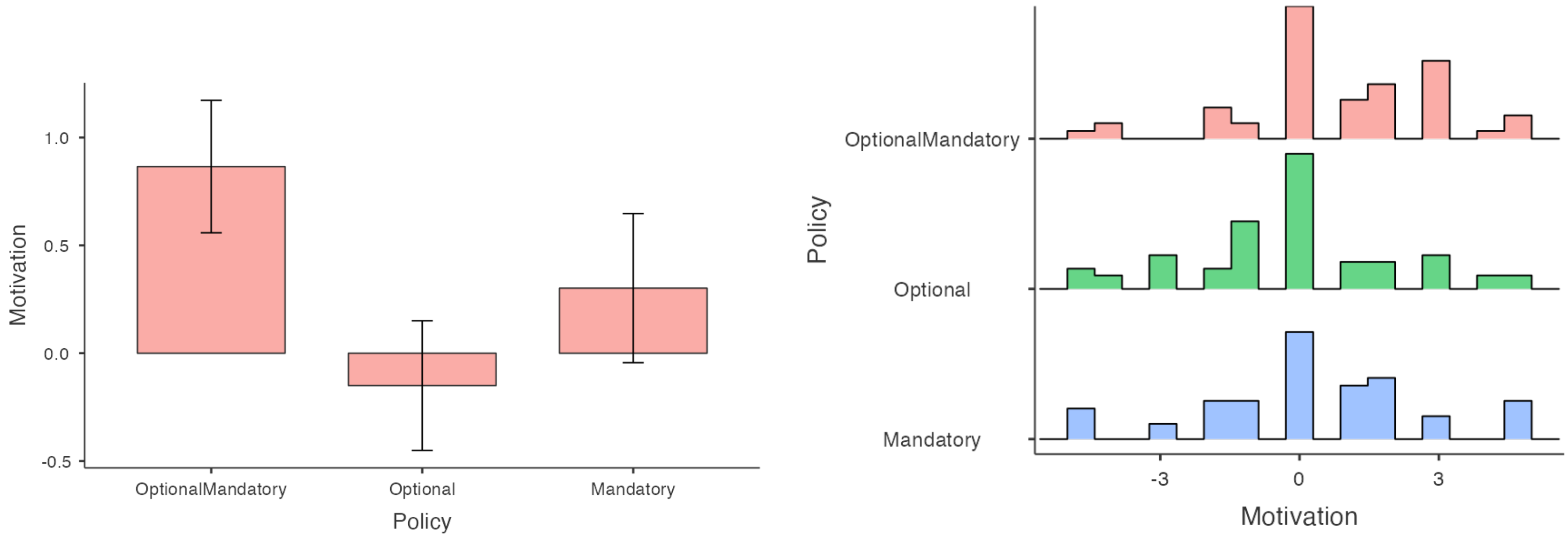

**Figure S3**

How did [POLICY] affect your learning in the class?

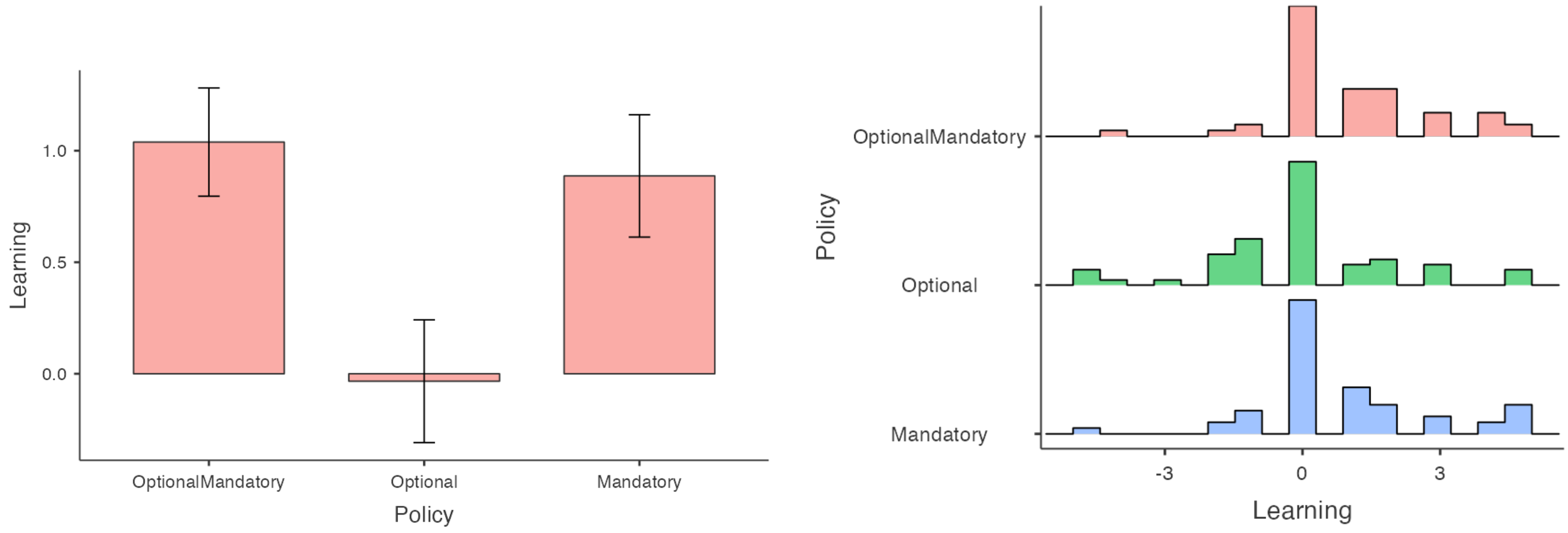

**Figure S4**

How did [POLICY] affect your relationship with your professor/teaching team?

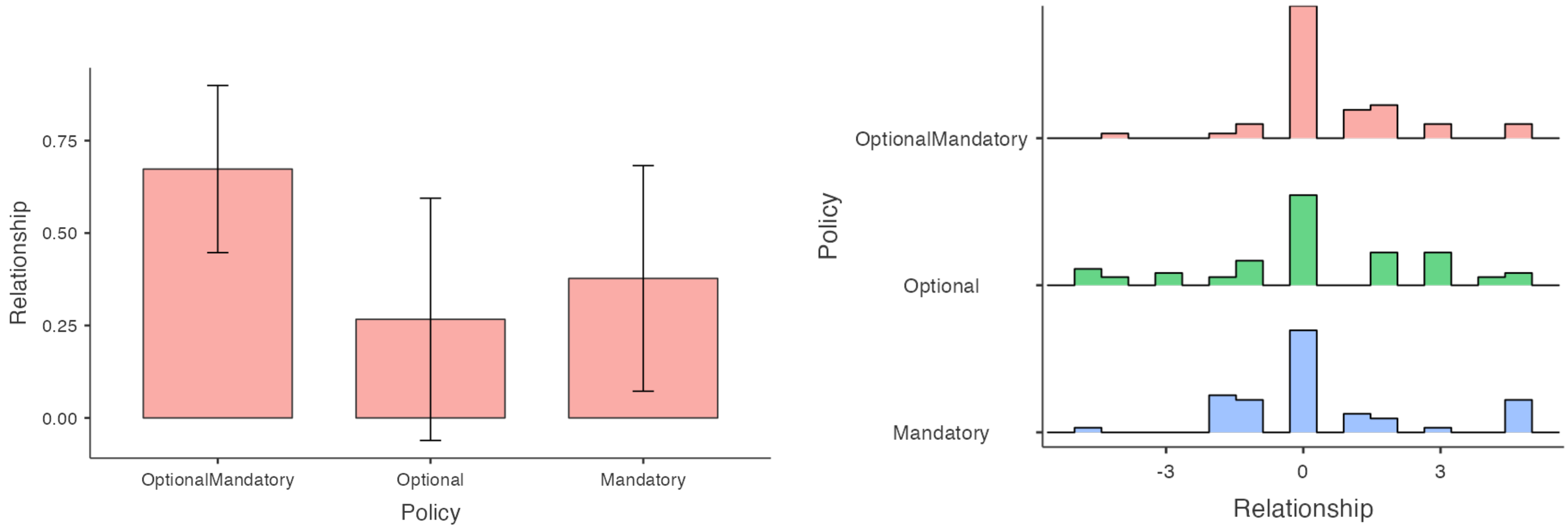

**Figure S5**

If you knew that another course you were considering taking had a [POLICY] policy, how would that affect the chances of you taking the course?

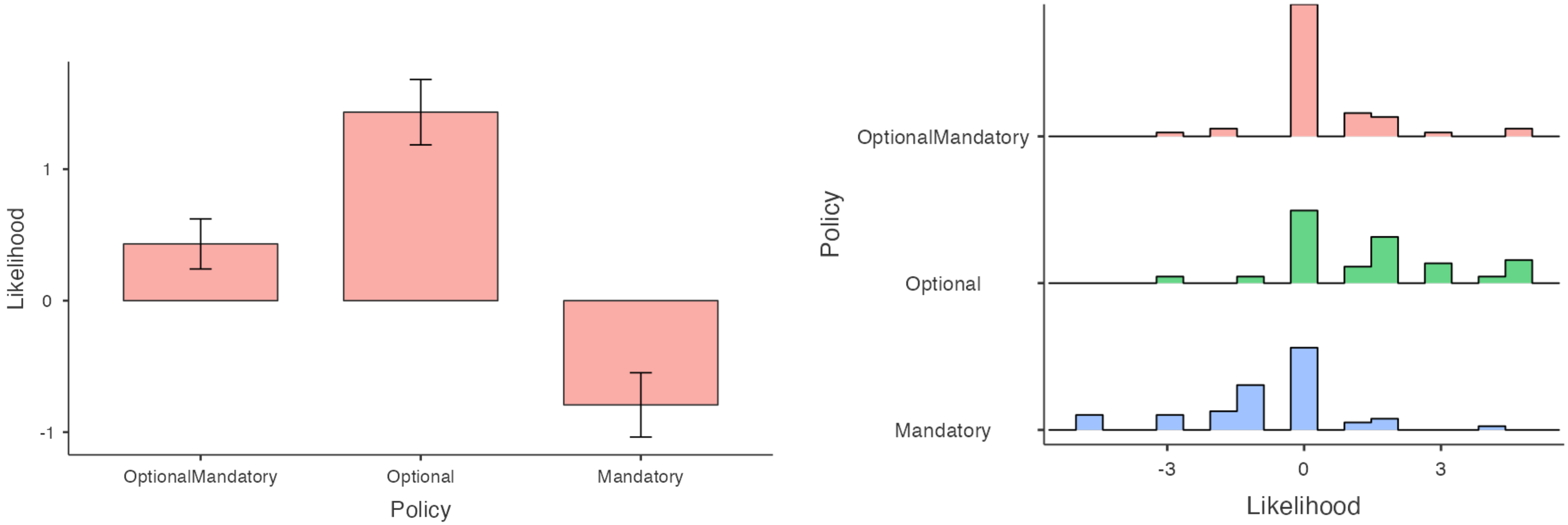

**Figure S6**

Overall, how would you evaluate the policy of [POLICY]?

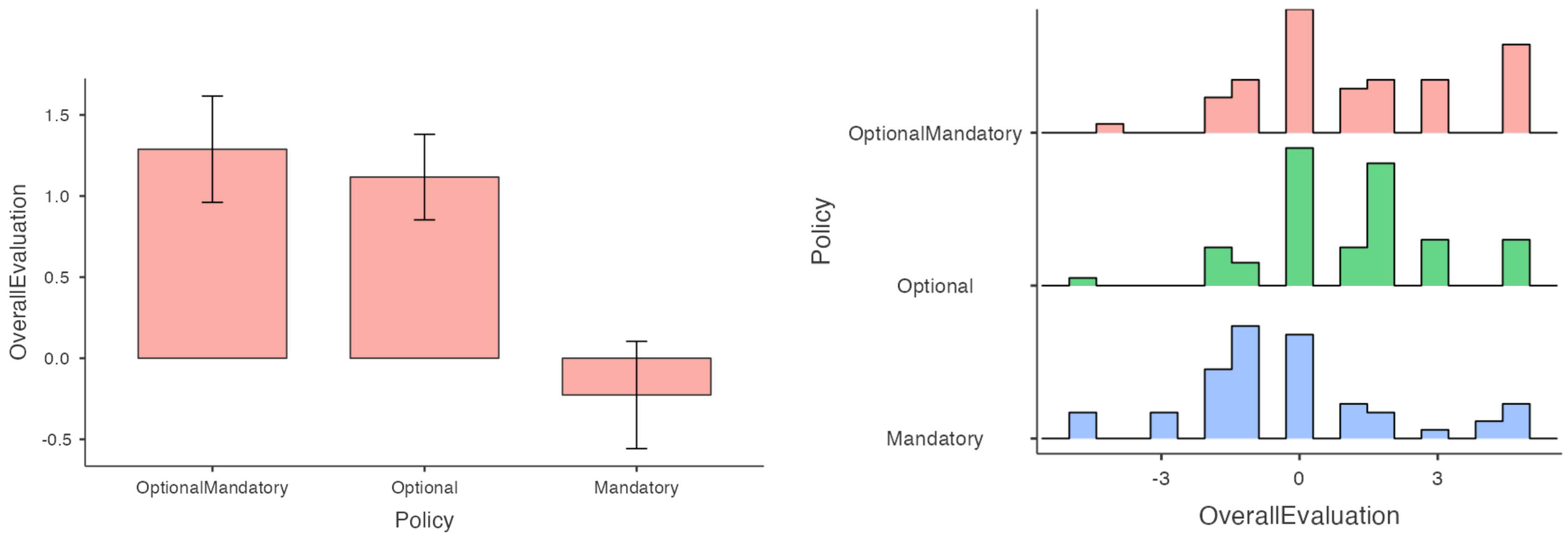

**Figure S7**

Overall, how do you think your peers would evaluate the policy of [POLICY]

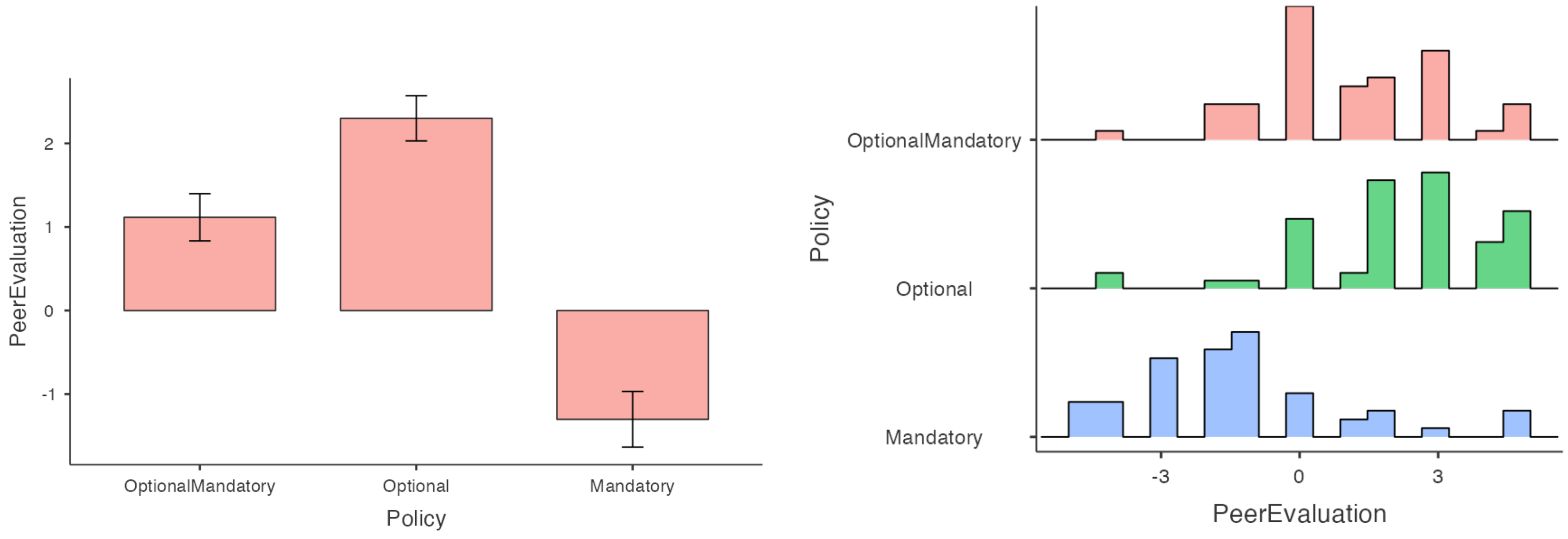

# Alternative Models (Study 1)

This is the fully crossed model reported in the text. Less complex models are presented below it.

# Generalized Mixed Model

Model Info

| Info | | |
|---|---|---|
| Model Type | Logistic Model | Model for binary y |
| Model | lme4::glmer | `P(Attended)` ~ 1 + `Attendance policy` + `Meeting number` + TeachingAssistant + `Attendance policy`:`Meeting number` + `Attendance policy`:TeachingAssistant + `Meeting number`:TeachingAssistant + `Meeting number`:TeachingAssistant:`Attendance policy` + ( 1 \| ID ) |
| Distribution | Binomial | Dichotomous event distribution of y |
| Link function | Logit | Log of the odd of y |
| Direction | P(y=1)/P(y=0) | P( P(Attended) = Present ) / P( P(Attended) = Absent ) |
| Sample size | 794 | |
| Converged | yes | |
| C.I. method | Wald | |

## Model Results

Model Fit

| Type | $R^2$ | df | LRT $X^2$ | p |
|---|---|---|---|---|
| Conditional | 0.31 | 12 | 105.46 | < .001 |
| Marginal | 0.08 | 11 | 27.82 | 0.003 |

Additional Indices

| Info | Model Value | Comment |
|---|---|---|
| LogLikelihood | -491.56 | |
| AIC | 1009.13 | Less is better |
| BIC | 1069.93 | Less is better |
| Deviance | 983.13 | Less is better |
| Residual DF | 781 | |
| Chi-squared/DF | 0.84 | Overdispersion indicator |

Fixed Effects Omnibus Tests

| | $X^2$ | df | p |
|---|---|---|---|
| Attendance policy | 3.24 | 1.00 | 0.072 |
| Meeting number | 9.31 | 1.00 | 0.002 |
| TeachingAssistant | 8.24 | 2.00 | 0.016 |
| Attendance policy ✻ Meeting number | 5.00 | 1.00 | 0.025 |
| Attendance policy ✻ TeachingAssistant | 0.72 | 2.00 | 0.698 |
| Meeting number ✻ TeachingAssistant | 1.81 | 2.00 | 0.405 |
| Attendance policy ✻ Meeting number ✻ TeachingAssistant | 2.56 | 2.00 | 0.278 |

Parameter Estimates (Fixed Coefficients)

| | | | | | Exp(B) 95% Confidence Intervals | | | |
|---|---|---|---|---|---|---|---|---|
| Names | Effect | Estimate | SE | Exp(B) | Lower | Upper | z | p |
| (Intercept) | (Intercept) | 0.29 | 0.14 | 1.34 | 1.01 | 1.77 | 2.02 | 0.044 |
| Attendance policy1 | Optional-Mandatory - Mandatory | 0.52 | 0.29 | 1.68 | 0.96 | 2.94 | 1.80 | 0.072 |
| Meeting number | Meeting number | -0.09 | 0.03 | 0.92 | 0.87 | 0.97 | -3.05 | 0.002 |
| TeachingAssistant1 | TA2 - TA1 | 0.97 | 0.34 | 2.65 | 1.35 | 5.21 | 2.82 | 0.005 |
| TeachingAssistant2 | TA3 - TA1 | 0.64 | 0.35 | 1.89 | 0.94 | 3.77 | 1.80 | 0.072 |
| Attendance policy1 ✻ Meeting number | (Optional-Mandatory - Mandatory) ✻ Meeting number | 0.13 | 0.06 | 1.14 | 1.02 | 1.27 | 2.24 | 0.025 |
| Attendance policy1 ✻ TeachingAssistant1 | (Optional-Mandatory - Mandatory) ✻ (TA2 - TA1) | -0.31 | 0.69 | 0.74 | 0.19 | 2.83 | -0.44 | 0.657 |
| Attendance policy1 ✻ TeachingAssistant2 | (Optional-Mandatory - Mandatory) ✻ (TA3 - TA1) | -0.60 | 0.71 | 0.55 | 0.14 | 2.20 | -0.85 | 0.397 |
| Meeting number ✻ TeachingAssistant1 | Meeting number ✻ (TA2 - TA1) | 0.09 | 0.07 | 1.10 | 0.96 | 1.26 | 1.32 | 0.186 |
| Meeting number ✻ TeachingAssistant2 | Meeting number ✻ (TA3 - TA1) | 0.06 | 0.07 | 1.06 | 0.93 | 1.22 | 0.86 | 0.391 |
| Attendance policy1 ✻ Meeting number ✻ TeachingAssistant1 | (Optional-Mandatory - Mandatory) ✻ Meeting number ✻ (TA2 - TA1) | -0.16 | 0.14 | 0.85 | 0.65 | 1.12 | -1.16 | 0.247 |
| Attendance policy1 ✻ Meeting number ✻ TeachingAssistant2 | (Optional-Mandatory - Mandatory) ✻ Meeting number ✻ (TA3 - TA1) | -0.22 | 0.14 | 0.81 | 0.61 | 1.06 | -1.53 | 0.126 |

Random Components

| Groups | Name | Variance | SD | ICC |
|---|---|---|---|---|
| ID | (Intercept) | 1.10 | 1.05 | 0.25 |
| Residual | | 3.29 | 1.81 | |

*Note.* Number of Obs: 794 , Number of groups: ID 82

Random Effect LRT

| Test | N. par | AIC | LRT | df | p |
|---|---|---|---|---|---|
| Intercept \| ID | 13.00 | 1009.13 | 63.46 | 1.00 | < .001 |

*Note.* No random coefficients specified. A generalized linear model is used for comparison.

### Post Hoc Tests

Post Hoc comparison: Attendance policy

| Comparison | | | | | 95% Confidence Intervals | | | |
|---|---|---|---|---|---|---|---|---|
| Attendance policy | vs | Attendance policy | OR | SE | Lower | Upper | z | $p_{bonferroni}$ |
| Mandatory | - | Optional-Mandatory | 0.60 | 0.17 | 0.26 | 0.93 | -1.80 | 0.072 |

### Simple Effects

ANOVA for Simple Effects of Meeting number

| Moderator | | | |
|---|---|---|---|
| Attendance policy | $X^2$ | df | p |
| Mandatory | 12.71 | 1.00 | < .001 |
| Optional-Mandatory | 0.37 | 1.00 | 0.541 |

Parameter Estimates for simple effects of Meeting number

| Moderator | | | | | Exp(B) 95% Confidence Intervals | | | |
|---|---|---|---|---|---|---|---|---|
| Attendance policy | Effect | Estimate | SE | Exp(B) | Lower | Upper | z | p |
| Mandatory | Meeting number | -0.15 | 0.04 | 0.86 | 0.79 | 0.93 | -3.56 | < .001 |
| Optional-Mandatory | Meeting number | -0.02 | 0.04 | 0.98 | 0.91 | 1.05 | -0.61 | 0.541 |

## Generalized Mixed Model

This model includes only the critical interaction term (Policy x Week)

Model Info

| Info | | |
|---|---|---|
| Model Type | Logistic Model | Model for binary y |
| Model | lme4::glmer | \`P(Attended)\` ~ 1 + \`Attendance policy\` + \`Meeting number\` + TeachingAssistant + \`Attendance policy\`:\`Meeting number\` + ( 1 \| ID ) |
| Distribution | Binomial | Dichotomous event distribution of y |
| Link function | Logit | Log of the odd of y |
| Direction | P(y=1)/P(y=0) | P( P(Attended) = Present ) / P( P(Attended) = Absent ) |
| Sample size | 794 | |
| Converged | yes | |
| C.I. method | Wald | |

## Model Results

Model Fit

| Type | $R^2$ | df | LRT $X^2$ | p |
|---|---|---|---|---|
| Conditional | 0.30 | 6 | 100.85 | < .001 |
| Marginal | 0.06 | 5 | 23.20 | < .001 |

Additional Indices

| Info | Model Value | Comment |
|---|---|---|
| LogLikelihood | -493.87 | |
| AIC | 1001.74 | Less is better |
| BIC | 1034.48 | Less is better |
| Deviance | 987.74 | Less is better |
| Residual DF | 787 | |
| Chi-squared/DF | 0.84 | Overdispersion indicator |

Fixed Effects Omnibus Tests

| | $X^2$ | df | p |
|---|---|---|---|
| Attendance policy | 3.15 | 1.00 | 0.076 |
| Meeting number | 9.30 | 1.00 | 0.002 |
| TeachingAssistant | 7.82 | 2.00 | 0.020 |
| Attendance policy ✻ Meeting number | 4.79 | 1.00 | 0.029 |

Parameter Estimates (Fixed Coefficients)

| Names | Effect | Estimate | SE | Exp(B) | Exp(B) 95% Confidence Intervals | | z | p |
|---|---|---|---|---|---|---|---|---|
| | | | | | Lower | Upper | | |
| (Intercept) | (Intercept) | 0.30 | 0.14 | 1.35 | 1.02 | 1.78 | 2.11 | 0.034 |
| Attendance policy1 | Optional-Mandatory - Mandatory | 0.51 | 0.28 | 1.66 | 0.95 | 2.90 | 1.78 | 0.076 |
| Meeting number | Meeting number | -0.09 | 0.03 | 0.92 | 0.87 | 0.97 | -3.05 | 0.002 |
| TeachingAssistant1 | TA2 - TA1 | 0.95 | 0.34 | 2.58 | 1.32 | 5.03 | 2.77 | 0.006 |
| TeachingAssistant2 | TA3 - TA1 | 0.58 | 0.35 | 1.79 | 0.90 | 3.54 | 1.67 | 0.095 |
| Attendance policy1 ✻ Meeting number | (Optional-Mandatory - Mandatory) ✻ Meeting number | 0.12 | 0.06 | 1.13 | 1.01 | 1.26 | 2.19 | 0.029 |

Random Components

| Groups | Name | Variance | SD | ICC |
|---|---|---|---|---|
| ID | (Intercept) | 1.09 | 1.04 | 0.25 |
| Residual | | 3.29 | 1.81 | |

*Note.* Number of Obs: 794 , Number of groups: ID 82

Random Effect LRT

| Test | N. par | AIC | LRT | df | p |
|---|---|---|---|---|---|
| Intercept \| ID | 7.00 | 1001.74 | 63.50 | 1.00 | < .001 |

*Note.* No random coefficients specified. A generalized linear model is used for comparison.

## Post Hoc Tests

Post Hoc comparison: Attendance policy

| Comparison | | | | | 95% Confidence Intervals | | | |
|---|---|---|---|---|---|---|---|---|
| Attendance policy | vs | Attendance policy | OR | SE | Lower | Upper | z | $p_{bonferroni}$ |
| Mandatory | - | Optional-Mandatory | 0.60 | 0.17 | 0.27 | 0.94 | -1.78 | 0.076 |

## Simple Effects

ANOVA for Simple Effects of Meeting number

| Moderator | | | |
|---|---|---|---|
| Attendance policy | $x^2$ | df | p |
| Mandatory | 12.60 | 1.00 | < .001 |
| Optional-Mandatory | 0.41 | 1.00 | 0.522 |

Parameter Estimates for simple effects of Meeting number

| Moderator | | | | | Exp(B) 95% Confidence Intervals | | | |
|---|---|---|---|---|---|---|---|---|
| Attendance policy | Effect | Estimate | SE | Exp(B) | Lower | Upper | z | p |
| Mandatory | Meeting number | -0.15 | 0.04 | 0.86 | 0.79 | 0.94 | -3.55 | < .001 |
| Optional-Mandatory | Meeting number | -0.02 | 0.04 | 0.98 | 0.91 | 1.05 | -0.64 | 0.522 |

## Generalized Mixed Model

This model leaves out the TA term entirely.

Model Info

| Info | | |
|---|---|---|
| Model Type | Logistic Model | Model for binary y |
| Model | lme4::glmer | `P(Attended)` ~ 1 + `Attendance policy` + `Meeting number` + `Attendance policy`:`Meeting number` + ( 1 \| ID ) |
| Distribution | Binomial | Dichotomous event distribution of y |
| Link function | Logit | Log of the odd of y |
| Direction | P(y=1)/P(y=0) | P( P(Attended) = Present ) / P( P(Attended) = Absent ) |
| Sample size | 794 | |
| Converged | yes | |
| C.I. method | Wald | |

### Model Results

Model Fit

| Type | $R^2$ | df | LRT $X^2$ | p |
|---|---|---|---|---|
| Conditional | 0.30 | 4 | 93.25 | < .001 |
| Marginal | 0.03 | 3 | 15.60 | 0.001 |

Additional Indices

| Info | Model Value | Comment |
|---|---|---|
| LogLikelihood | -497.67 | |
| AIC | 1005.35 | Less is better |
| BIC | 1028.73 | Less is better |
| Deviance | 995.35 | Less is better |
| Residual DF | 789 | |
| Chi-squared/DF | 0.83 | Overdispersion indicator |

Fixed Effects Omnibus Tests

| | $X^2$ | df | p |
|---|---|---|---|
| Attendance policy | 2.34 | 1.00 | 0.126 |
| Meeting number | 9.10 | 1.00 | 0.003 |
| Attendance policy ✻ Meeting number | 4.63 | 1.00 | 0.031 |

Parameter Estimates (Fixed Coefficients)

| | | | | | Exp(B) 95% Confidence Intervals | | | |
|---|---|---|---|---|---|---|---|---|
| Names | Effect | Estimate | SE | Exp(B) | Lower | Upper | z | p |
| (Intercept) | (Intercept) | 0.29 | 0.15 | 1.34 | 1.00 | 1.79 | 1.96 | 0.050 |
| Attendance policy1 | Optional-Mandatory - Mandatory | 0.45 | 0.30 | 1.57 | 0.88 | 2.81 | 1.53 | 0.126 |
| Meeting number | Meeting number | -0.09 | 0.03 | 0.92 | 0.87 | 0.97 | -3.02 | 0.003 |
| Attendance policy1 ✻ Meeting number | (Optional-Mandatory - Mandatory) ✻ Meeting number | 0.12 | 0.06 | 1.13 | 1.01 | 1.26 | 2.15 | 0.031 |

Random Components

| Groups | Name | Variance | SD | ICC |
|---|---|---|---|---|
| ID | (Intercept) | 1.24 | 1.11 | 0.27 |
| Residual | | 3.29 | 1.81 | |

*Note.* Number of Obs: 794 , Number of groups: ID 82

Random Effect LRT

| Test | N. par | AIC | LRT | df | p |
|---|---|---|---|---|---|
| Intercept \| ID | 5.00 | 1005.35 | 75.70 | 1.00 | < .001 |

*Note.* No random coefficients specified. A generalized linear model is used for comparison.

## Post Hoc Tests

Post Hoc comparison: Attendance policy

| Comparison | | | | | 95% Confidence Intervals | | | |
|---|---|---|---|---|---|---|---|---|
| Attendance policy | vs | Attendance policy | OR | SE | Lower | Upper | z | $p_{bonferroni}$ |
| Mandatory | - | Optional-Mandatory | 0.64 | 0.19 | 0.27 | 1.00 | -1.53 | 0.126 |

## Simple Effects

ANOVA for Simple Effects of Meeting number

| Moderator | | | |
|---|---|---|---|
| Attendance policy | $x^2$ | df | p |
| Mandatory | 12.30 | 1.00 | < .001 |
| Optional-Mandatory | 0.41 | 1.00 | 0.521 |

Parameter Estimates for simple effects of Meeting number

| Moderator | | | | | Exp(B) 95% Confidence Intervals | | | |
|---|---|---|---|---|---|---|---|---|
| Attendance policy | Effect | Estimate | SE | Exp(B) | Lower | Upper | z | p |
| Mandatory | Meeting number | -0.15 | 0.04 | 0.86 | 0.80 | 0.94 | -3.51 | < .001 |
| Optional-Mandatory | Meeting number | -0.02 | 0.04 | 0.98 | 0.91 | 1.05 | -0.64 | 0.521 |

# Comparison of Cohorts from Study 2 of 'Choosing to Learn: The Value of Student Autonomy in Post-Secondary Education'

## Home department

Contingency Tables

| | Cohort | | |
|---|---|---|---|
| **Department** | **FreeToSwitch** | **Mandatory** | **Total** |
| ARC | 1 | 0 | 1 |
| BA | 3 | 5 | 8 |
| BSC | 2 | 0 | 2 |
| C00 | 6 | 5 | 11 |
| CEE | 1 | 0 | 1 |
| CHE | 1 | 0 | 1 |
| CMY | 0 | 1 | 1 |
| CS | 11 | 3 | 14 |
| DRA | 1 | 0 | 1 |
| ECE | 5 | 5 | 10 |
| H00 | 10 | 2 | 12 |
| HSS | 9 | 3 | 12 |
| M00 | 7 | 4 | 11 |
| MEG | 1 | 3 | 4 |
| MSC | 4 | 2 | 6 |
| MUS | 2 | 1 | 3 |
| PHY | 1 | 1 | 2 |
| PSY | 1 | 1 | 2 |
| QBS | 1 | 0 | 1 |
| SCS | 1 | 0 | 1 |
| SDS | 1 | 0 | 1 |
| SHS | 0 | 1 | 1 |
| STA | 7 | 4 | 11 |
| Z00 | 4 | 1 | 5 |
| Total | 80 | 42 | 122 |

$\chi^2$ Tests

| | Value | df | p |
|---|---|---|---|
| $\chi^2$ | 20.04 | 23 | 0.639 |
| N | 122 | | |

Gamma

| | | 95% Confidence Intervals | |
|---|---|---|---|
| Gamma | Standard Error | Lower | Upper |
| -0.04 | 0.12 | -0.28 | 0.20 |

## Home college

Contingency Tables

| | Cohort | | |
|---|---|---|---|
| College | FreeToSwitch | Mandatory | Total |
| CFA | 4 | 1 | 5 |
| CIT | 14 | 13 | 27 |
| CMU | 1 | 1 | 2 |
| DC | 28 | 10 | 38 |
| MCS | 14 | 8 | 22 |
| SCS | 16 | 4 | 20 |
| TSB | 3 | 5 | 8 |
| Total | 80 | 42 | 122 |

$x^2$ Tests

| | Value | df | p |
|---|---|---|---|
| $x^2$ | 8.71 | 6 | 0.191 |
| N | 122 | | |

Gamma

| | | 95% Confidence Intervals | |
|---|---|---|---|
| Gamma | Standard Error | Lower | Upper |
| -0.06 | 0.14 | -0.33 | 0.21 |

## Major

Contingency Tables

| Major | Cohort | | |
|---|---|---|---|
| | FreeToSwitch | Mandatory | Total |
| AI | 1 | 0 | 1 |
| ARC | 1 | 0 | 1 |
| BA | 3 | 5 | 8 |
| BSC | 1 | 0 | 1 |
| CHE | 1 | 0 | 1 |
| CIV | 1 | 0 | 1 |
| CMY | 0 | 1 | 1 |
| COMPFIN | 0 | 1 | 1 |
| CS | 11 | 3 | 14 |
| DECSCI | 1 | 0 | 1 |
| DRA | 1 | 0 | 1 |
| ECE | 5 | 5 | 10 |
| ECOSTA | 1 | 0 | 1 |
| INFOSYS | 9 | 2 | 11 |
| MEG | 1 | 3 | 4 |
| MPEU | 1 | 0 | 1 |
| MPOBO | 0 | 1 | 1 |
| MSC | 3 | 0 | 3 |
| MSCDML | 1 | 1 | 2 |
| MSCSTA | 0 | 1 | 1 |
| NEUROSCI | 2 | 0 | 2 |
| PHY | 1 | 0 | 1 |
| PHYBSC | 0 | 1 | 1 |
| PSY | 1 | 1 | 2 |
| STA | 1 | 1 | 2 |
| STAMACH | 5 | 3 | 8 |
| UNDECLARED | 28 | 13 | 41 |
| Total | 80 | 42 | 122 |

$\chi^2$ Tests

| | Value | df | p |
|---|---|---|---|
| $\chi^2$ | 27.33 | 26 | 0.392 |
| N | 122 | | |

Gamma

| Gamma | Standard Error | 95% Confidence Intervals | |
|---|---|---|---|
| | | Lower | Upper |
| -0.04 | 0.13 | -0.28 | 0.21 |

# Year in college

Contingency Tables

| | Cohort | | |
|---|---|---|---|
| Class | FreeToSwitch | Mandatory | Total |
| 1 | 29 | 16 | 45 |
| 2 | 30 | 11 | 41 |
| 3 | 12 | 9 | 21 |
| 4 | 9 | 5 | 14 |
| Total | 80 | 41 | 121 |

$\chi^2$ Tests

| | Value | df | p |
|---|---|---|---|
| $\chi^2$ | 1.74 | 3 | 0.627 |
| N | 121 | | |

Gamma

| | | 95% Confidence Intervals | |
|---|---|---|---|
| Gamma | Standard Error | Lower | Upper |
| 0.03 | 0.15 | -0.26 | 0.33 |

# Complete models for Study 2 from 'Choosing to Learn: The Value of Student

## Student effort (original scale)

This is the model presented in the text. The DV has six levels like the scale students used t report their time on assignments. Because so few submissions reported taking over 3 hrs, estimates for the higher thresholds can be less reliable. Beneath this model are the results of binning the higher time categories into one.

Model Info

| Info | | |
|---|---|---|
| Model Type | Cumlative Link Model | Proportional odds logistic |
| Model | ordinal::clmm | \`Time on homework\` ~ 1 + \`Homework number\` + Cohort + Cohort:\`Homework number\` + ( 1 \| ID ) |
| Distribution | Logistic | |
| Link function | Logit | Log of the odd of y |
| Direction | P(Y ≤ j)/P(Y > j) | j= <1 hr \| 1-2 hrs \| 2-3 hrs \| 3-4 hrs \| 4-5 hrs \| >5 hrs |
| Sample size | 1036 | |
| Converged | yes | |
| C.I. method | Wald | |

### Model Results

Model Fit

| Type | $R^2$ | df | LRT $X^2$ | p |
|---|---|---|---|---|
| Conditional | 0.438 | 4.000 | 342.434 | < .001 |
| Marginal | 0.080 | 3.000 | 30.823 | < .001 |

Additional Indices

| Info | Model Value | Comment |
|---|---|---|
| LogLikelihood | -1310.807 | |
| AIC | 2639.613 | Less is better |
| BIC | 2684.102 | Less is better |
| Deviance | 6933.342 | Less is better |

Fixed Effects Omnibus Tests

| | $X^2$ | df | p |
|---|---|---|---|
| Homework number | 14.677 | 1.000 | < .001 |
| Cohort | 15.288 | 1.000 | < .001 |
| Homework number ✻ Cohort | 0.006 | 1.000 | 0.937 |

Parameter Estimates (Fixed Coefficients)

| | | | | | Exp(B) 95% Confidence Intervals | | | |
|---|---|---|---|---|---|---|---|---|
| Names | Effect | Estimate | SE | Exp(B) | Lower | Upper | z | p |
| (Threshold) | <1 hr\|1-2 hrs | -2.949 | 0.201 | 0.052 | 0.035 | 0.078 | -14.682 | < .001 |
| (Threshold) | 1-2 hrs\|2-3 hrs | 0.014 | 0.170 | 1.014 | 0.727 | 1.413 | 0.081 | 0.936 |
| (Threshold) | 2-3 hrs\|3-4 hrs | 2.165 | 0.185 | 8.711 | 6.060 | 12.520 | 11.694 | < .001 |
| (Threshold) | 3-4 hrs\|4-5 hrs | 3.676 | 0.220 | 39.488 | 25.643 | 60.807 | 16.689 | < .001 |
| (Threshold) | 4-5 hrs\|>5 hrs | 5.646 | 0.339 | 283.262 | 145.617 | 551.016 | 16.632 | < .001 |
| Homework number | Homework number | -0.071 | 0.018 | 0.932 | 0.899 | 0.966 | -3.831 | < .001 |
| Cohort1 | FreeToSwitch - Mandatory | 1.289 | 0.330 | 3.628 | 1.902 | 6.923 | 3.910 | < .001 |
| Homework number ✻ Cohort1 | Homework number:Cohort1 | 0.003 | 0.037 | 1.003 | 0.934 | 1.077 | 0.080 | 0.937 |

Random Components

| Groups | Name | Variance | SD | ICC |
|---|---|---|---|---|
| ID | (Intercept) | 2.097 | 1.448 | 0.389 |
| Residual | | 3.290 | 1.814 | |

*Note.* Number of Obs: 1036 , Number of groups: ID 98

Random Effect LRT

| Test | N. par | AIC | LRT | df | p |
|---|---|---|---|---|---|
| Intercept \| ID | 9.000 | 2911.642 | 272.029 | 1.000 | < .001 |

*Note.* No random coefficients in the nested model. A fixed effects ordinal model is used for comparison

### Simple Effects

ANOVA for Simple Effects of Homework number

| Moderator | | | |
|---|---|---|---|
| Cohort | $x^2$ | df | p |
| Mandatory | 6.237 | 1.000 | 0.013 |
| FreeToSwitch | 9.259 | 1.000 | 0.002 |

Parameter Estimates for simple effects of Homework number

| Moderator | | | | | Exp(B) 95% Confidence Intervals | | | |
|---|---|---|---|---|---|---|---|---|
| Cohort | Effect | Estimate | SE | Exp(B) | Lower | Upper | z | p |
| Mandatory | Homework number | -0.072 | 0.029 | 0.930 | 0.879 | 0.985 | -2.497 | 0.013 |
| FreeToSwitch | Homework number | -0.069 | 0.023 | 0.933 | 0.893 | 0.976 | -3.043 | 0.002 |

### Estimated Marginal Means

Estimate Marginal Means - Cohort

| | | | 95% Confidence Intervals | |
|---|---|---|---|---|
| Cohort | Mean | SE | Lower | Upper |
| Mandatory | 2.322 | 0.097 | 2.132 | 2.513 |
| FreeToSwitch | 2.858 | 0.091 | 2.680 | 3.036 |

*Note.* Expected means are expressed as expected class
*Note.* Classes are: 1=<1 hr, 2=1-2 hrs, 3=2-3 hrs, 4=3-4 hrs, 5=4-5 hrs, 6=>5 hrs

Note: Classes are: 1=<1 hr, 2=1-2 hrs, 3=2-3 hrs, 4=3-4 hrs, 5=4-5 hrs, 6=>5 hrs

## Student effort (binned scale)

Because very few submissions reported taking longer than 4 hrs, we report this model which bins submissions that reported 3-4 hrs, 4-5 hrs, and >5 hrs.

Model Info

| Info | | |
|---|---|---|
| Model Type | Cumlative Link Model | Proportional odds logistic |
| Model | ordinal::clmm | \`Time on homework (binned)\` ~ 1 + \`Homework number\` + Cohort + Cohort:\`Homework number\` + ( 1 \| ID ) |
| Distribution | Logistic | |
| Link function | Logit | Log of the odd of y |
| Direction | P(Y ≤ j)/P(Y > j) | j= <1 hr \| 1-2 hrs \| 2-3 hrs \| >3 hrs |
| Sample size | 1036 | |
| Converged | yes | |
| C.I. method | Wald | |

### Model Results

Model Fit

| Type | $R^2$ | df | LRT $X^2$ | p |
|---|---|---|---|---|
| Conditional | 0.428 | 4.000 | 319.672 | < .001 |
| Marginal | 0.075 | 3.000 | 28.437 | < .001 |

Additional Indices

| Info | Model Value | Comment |
|---|---|---|
| LogLikelihood | -1151.946 | |
| AIC | 2317.892 | Less is better |
| BIC | 2352.494 | Less is better |
| Deviance | 5811.899 | Less is better |

Fixed Effects Omnibus Tests

| | $X^2$ | df | p |
|---|---|---|---|
| Homework number | 13.403 | 1.000 | < .001 |
| Cohort | 14.587 | 1.000 | < .001 |
| Homework number ✻ Cohort | 0.055 | 1.000 | 0.814 |

Parameter Estimates (Fixed Coefficients)

| | | | | | Exp(B) 95% Confidence Intervals | | | |
|---|---|---|---|---|---|---|---|---|
| Names | Effect | Estimate | SE | Exp(B) | Lower | Upper | z | p |
| (Threshold) | <1 hr\|1-2 hrs | -2.940 | 0.199 | 0.053 | 0.036 | 0.078 | -14.739 | < .001 |
| (Threshold) | 1-2 hrs\|2-3 hrs | 0.017 | 0.167 | 1.018 | 0.733 | 1.412 | 0.105 | 0.917 |
| (Threshold) | 2-3 hrs\|>3 hrs | 2.163 | 0.183 | 8.699 | 6.083 | 12.442 | 11.850 | < .001 |
| Homework number | Homework number | -0.069 | 0.019 | 0.933 | 0.900 | 0.968 | -3.661 | < .001 |
| Cohort1 | FreeToSwitch - Mandatory | 1.242 | 0.325 | 3.462 | 1.830 | 6.548 | 3.819 | < .001 |
| Homework number ✻ Cohort1 | Homework number:Cohort1 | 0.009 | 0.037 | 1.009 | 0.938 | 1.085 | 0.235 | 0.814 |

Random Components

| Groups | Name | Variance | SD | ICC |
|---|---|---|---|---|
| ID | (Intercept) | 2.033 | 1.426 | 0.382 |
| Residual | | 3.290 | 1.814 | |

*Note.* Number of Obs: 1036 , Number of groups: ID 98

Random Effect LRT

| Test | N. par | AIC | LRT | df | p |
|---|---|---|---|---|---|
| Intercept \| ID | 7.000 | 2568.665 | 250.773 | 1.000 | < .001 |

*Note.* No random coefficients in the nested model. A fixed effects ordinal model is used for comparison

## Simple Effects

ANOVA for Simple Effects of Homework number

| Moderator | | | |
|---|---|---|---|
| Cohort | $x^2$ | df | p |
| Mandatory | 6.310 | 1.000 | 0.012 |
| FreeToSwitch | 7.491 | 1.000 | 0.006 |

Parameter Estimates for simple effects of Homework number

| Moderator | | | | | Exp(B) 95% Confidence Intervals | | | |
|---|---|---|---|---|---|---|---|---|
| Cohort | Effect | Estimate | SE | Exp(B) | Lower | Upper | z | p |
| Mandatory | Homework number | -0.073 | 0.029 | 0.929 | 0.878 | 0.984 | -2.512 | 0.012 |
| FreeToSwitch | Homework number | -0.065 | 0.024 | 0.938 | 0.895 | 0.982 | -2.737 | 0.006 |

## Estimated Marginal Means

Estimate Marginal Means - Cohort

| | | | 95% Confidence Intervals | |
|---|---|---|---|---|
| Cohort | Mean | SE | Lower | Upper |
| Mandatory | 2.314 | 0.092 | 2.134 | 2.495 |
| FreeToSwitch | 2.795 | 0.080 | 2.638 | 2.952 |

*Note.* Expected means are expressed as expected class
*Note.* Classes are: 1=<1 hr, 2=1-2 hrs, 3=2-3 hrs, 4=>3 hrs

Note: Classes are: 1=<1 hr, 2=1-2 hrs, 3=2-3 hrs, 4=>3 hrs

# Student performance (logistic mixed model)

This is the model presented in the text. This analysis excludes the 0.16% of submissions which received a '0' in the free-to-switch cohort and corresponds to what we report in the main text. This decision allows us to employ a logistic mixed effects model, which is somewhat more straightforward to interpret.

Model Info

| Info | | |
|---|---|---|
| Model Type | Logistic Model | Model for binary y |
| Model | lme4::glmer | \`Grade (binary)\` ~ 1 + \`Homework number\` + Cohort + Cohort:\`Homework number\` + ( 1 \| ID ) |
| Distribution | Binomial | Dichotomous event distribution of y |
| Link function | Logit | Log of the odd of y |
| Direction | P(y=1)/P(y=0) | P( Grade (binary) = 2 ) / P( Grade (binary) = 1 ) |
| Sample size | 1023 | |
| Converged | yes | |
| C.I. method | Wald | |

## Model Results

Model Fit

| Type | $R^2$ | df | LRT $X^2$ | p |
|---|---|---|---|---|
| Conditional | 0.243 | 4 | 86.780 | < .001 |
| Marginal | 0.099 | 3 | 45.661 | < .001 |

Additional Indices

| Info | Model Value | Comment |
|---|---|---|
| LogLikelihood | -567.458 | |
| AIC | 1144.916 | Less is better |
| BIC | 1169.568 | Less is better |
| Deviance | 1134.916 | Less is better |
| Residual DF | 1018 | |
| Chi-squared/DF | 0.863 | Overdispersion indicator |

Fixed Effects Omnibus Tests

| | $X^2$ | df | p |
|---|---|---|---|
| Homework number | 19.635 | 1.000 | < .001 |
| Cohort | 17.960 | 1.000 | < .001 |
| Homework number ✻ Cohort | 10.496 | 1.000 | 0.001 |

Parameter Estimates (Fixed Coefficients)

| | | | | | Exp(B) 95% Confidence Intervals | | | |
|---|---|---|---|---|---|---|---|---|
| Names | Effect | Estimate | SE | Exp(B) | Lower | Upper | z | p |
| (Intercept) | (Intercept) | 1.046 | 0.118 | 2.846 | 2.260 | 3.584 | 8.894 | < .001 |
| Homework number | Homework number | 0.101 | 0.023 | 1.106 | 1.058 | 1.157 | 4.431 | < .001 |
| Cohort1 | FreeToSwitch - Mandatory | 0.971 | 0.229 | 2.642 | 1.686 | 4.140 | 4.238 | < .001 |
| Homework number ✻ Cohort1 | Homework number ✻ (FreeToSwitch - Mandatory) | 0.147 | 0.045 | 1.159 | 1.060 | 1.266 | 3.240 | 0.001 |

Random Components

| Groups | Name | Variance | SD | ICC |
|---|---|---|---|---|
| ID | (Intercept) | 0.624 | 0.790 | 0.159 |
| Residual | | 3.290 | 1.814 | |

*Note.* Number of Obs: 1023 , Number of groups: ID 98

Random Effect LRT

| Test | N. par | AIC | LRT | df | p |
|---|---|---|---|---|---|
| Intercept \| ID | 5.000 | 1144.916 | 28.780 | 1.000 | < .001 |

*Note.* No random coefficients specified. A generalized linear model is used for comparison.

## Simple Effects

ANOVA for Simple Effects of Homework number

| Moderator | | | |
|---|---|---|---|
| Cohort | $x^2$ | df | p |
| Mandatory | 0.760 | 1.000 | 0.383 |
| FreeToSwitch | 28.071 | 1.000 | < .001 |

Parameter Estimates for simple effects of Homework number

| Moderator | | | | | Exp(B) 95% Confidence Intervals | | | |
|---|---|---|---|---|---|---|---|---|
| Cohort | Effect | Estimate | SE | Exp(B) | Lower | Upper | z | p |
| Mandatory | Homework number | 0.027 | 0.031 | 1.028 | 0.966 | 1.093 | 0.872 | 0.383 |
| FreeToSwitch | Homework number | 0.175 | 0.033 | 1.191 | 1.116 | 1.270 | 5.298 | < .001 |

### Estimated Marginal Means

Estimate Marginal Means - Cohort

| | | | 95% Confidence Intervals | |
|---|---|---|---|---|
| Cohort | Mean | SE | Lower | Upper |
| Mandatory | 0.637 | 0.039 | 0.556 | 0.710 |
| FreeToSwitch | 0.822 | 0.023 | 0.773 | 0.863 |

*Note*. Expected means are expressed as probabilities

## Student performance (ordinal mixed model)

This analysis includes the 0.16% of submissions which received a '0' in the free-to-switch cohort. We present this model to show that our results do not depend on the decision to exclude those submissions and use a logistic model.

Model Info

| Info | | |
|---|---|---|
| Model Type | Cumlative Link Model | Proportional odds logistic |
| Model | ordinal::clmm | Grade ~ 1 + \`Homework number\` + Cohort + Cohort:\`Homework number\` + ( 1 \| ID ) |
| Distribution | Logistic | |
| Link function | Logit | Log of the odd of y |
| Direction | P(Y ≤ j)/P(Y > j) | j= 0 \| 1 \| 2 |
| Sample size | 1031 | |
| Converged | yes | |
| C.I. method | Wald | |

### Model Results

Model Fit

| Type | $R^2$ | df | LRT $X^2$ | p |
|---|---|---|---|---|
| Conditional | 0.259 | 4.000 | 90.687 | < .001 |
| Marginal | 0.080 | 3.000 | 38.978 | < .001 |

Additional Indices

| Info | Model Value | Comment |
|---|---|---|
| LogLikelihood | -623.880 | |
| AIC | 1259.760 | Less is better |
| BIC | 1289.390 | Less is better |
| Deviance | 4442.864 | Less is better |

Fixed Effects Omnibus Tests

| | $X^2$ | df | p |
|---|---|---|---|
| Homework number | 18.459 | 1.000 | < .001 |
| Cohort | 11.618 | 1.000 | < .001 |
| Homework number ✻ Cohort | 9.757 | 1.000 | 0.002 |

Parameter Estimates (Fixed Coefficients)

| Names | Effect | Estimate | SE | Exp(B) | Exp(B) 95% Confidence Intervals Lower | Upper | z | p |
|---|---|---|---|---|---|---|---|---|
| (Threshold) | 0\|1 | -5.070 | 0.346 | 0.006 | 0.003 | 0.012 | -14.657 | < .001 |
| (Threshold) | 1\|2 | -1.014 | 0.125 | 0.363 | 0.284 | 0.464 | -8.090 | < .001 |
| Homework number | Homework number | 0.097 | 0.023 | 1.102 | 1.054 | 1.151 | 4.296 | < .001 |
| Cohort1 | FreeToSwitch - Mandatory | 0.831 | 0.245 | 2.297 | 1.422 | 3.710 | 3.398 | < .001 |
| Homework number ✻ Cohort1 | Homework number:Cohort1 | 0.140 | 0.045 | 1.151 | 1.054 | 1.256 | 3.124 | 0.002 |

Random Components

| Groups | Name | Variance | SD | ICC |
|---|---|---|---|---|
| ID | (Intercept) | 0.794 | 0.891 | 0.194 |
| Residual | | 3.290 | 1.814 | |

*Note.* Number of Obs: 1031 , Number of groups: ID 98

Random Effect LRT

| Test | N. par | AIC | LRT | df | p |
|---|---|---|---|---|---|
| Intercept \| ID | 6.000 | 1302.040 | 42.280 | 1.000 | < .001 |

*Note.* No random coefficients in the nested model. A fixed effects ordinal model is used for comparison

## Simple Effects

ANOVA for Simple Effects of Homework number

| Moderator Cohort | $x^2$ | df | p |
|---|---|---|---|
| Mandatory | 0.721 | 1.000 | 0.396 |
| FreeToSwitch | 26.734 | 1.000 | < .001 |

Parameter Estimates for simple effects of Homework number

| Moderator Cohort | Effect | Estimate | SE | Exp(B) | Exp(B) 95% Confidence Intervals Lower | Upper | z | p |
|---|---|---|---|---|---|---|---|---|
| Mandatory | Homework number | 0.027 | 0.031 | 1.027 | 0.966 | 1.092 | 0.849 | 0.396 |
| FreeToSwitch | Homework number | 0.167 | 0.032 | 1.182 | 1.109 | 1.259 | 5.170 | < .001 |

## Estimated Marginal Means

Estimate Marginal Means - Cohort

| Cohort | Mean | SE | 95% Confidence Intervals Lower | Upper |
|---|---|---|---|---|
| Mandatory | 2.636 | 0.044 | 2.550 | 2.722 |
| FreeToSwitch | 2.803 | 0.027 | 2.751 | 2.855 |

*Note.* Expected means are expressed as expected class
*Note.* Classes are: 1=0, 2=1, 3=2

Note: Classes are: 1=0, 2=1, 3=2
